# Unveiling Novel miRNA–mRNA Interactions and Their Prognostic Roles in Triple-Negative Breast Cancer: Insights into miR-210, miR-183, miR-21, and miR-181b

**DOI:** 10.3390/ijms26051916

**Published:** 2025-02-23

**Authors:** Jiatong Xu, Xiaoxuan Cai, Junyang Huang, Hsi-Yuan Huang, Yong-Fei Wang, Xiang Ji, Yuxin Huang, Jie Ni, Huali Zuo, Shangfu Li, Yang-Chi-Dung Lin, Hsien-Da Huang

**Affiliations:** 1 School of Medicine, The Chinese University of Hong Kong, Shenzhen 518172, China; 117010335@link.cuhk.edu.cn (J.X.); xiaoxuancai@link.cuhk.edu.cn (X.C.); junyanghuang@link.cuhk.edu.cn (J.H.); huanghsiyuan@cuhk.edu.cn (H.-Y.H.); yfwang@cuhk.edu.cn (Y.-F.W.); xiangji@link.cuhk.edu.cn (X.J.); yuxinhuang@link.cuhk.edu.cn (Y.H.); jennyni@cuhk.edu.cn (J.N.); zuohuali@cuhk.edu.cn (H.Z.); lishangfu@cuhk.edu.cn (S.L.); 2 Warshel Institute for Computational Biology, School of Medicine, The Chinese University of Hong Kong, Shenzhen 518172, China; 3 Guangdong Provincial Key Laboratory of Digital Biology and Drug Development, The Chinese University of Hong Kong, Shenzhen 518172, China

**Keywords:** triple-negative breast cancer, miRNA–target interaction, machine learning, regulatory network, prognosis

## Abstract

Triple-negative breast cancer (TNBC) poses a major clinical challenge due to its aggressive progression and limited treatment options, making early diagnosis and prognosis critical. MicroRNAs (miRNAs) are crucial post-transcriptional regulators that influence gene expression. In this study, we unveil novel miRNA–mRNA interactions and introduce a prognostic model based on miRNA–target interaction (MTI), integrating miRNA–mRNA regulatory correlation inference and the machine learning method to effectively predict the survival outcomes in TNBC cohorts. Using this method, we identified four key miRNAs (miR-181b-5p, miR-21-5p, miR-210-3p, miR-183-5p) targeting eight downstream target genes, forming a novel regulatory network of 19 validated miRNA–mRNA pairs. A prognostic model constructed based on the top 10 significant MTI pairs using random forest combination effectively classified patient survival outcomes in both TCGA and independent dataset GSE19783 cohorts, demonstrating good predictive accuracy and valuable prognostic insights for TNBC patients. Further analysis uncovered a complex network of 71 coherent feed-forward loops involving transcription factors, miRNAs, and target genes, shedding light on the mechanisms driving TNBC progression. This study underscores the importance of considering regulatory networks in cancer prognosis and provides a foundation for new therapeutic strategies aimed at improving TNBC treatment outcomes.

## 1. Introduction

Breast cancer, a prevalent malignancy in females, includes triple-negative breast cancer (TNBC) as its most aggressive subtype. TNBC is defined by the absence of estrogen receptor (ER), progesterone receptor (PR), and human epidermal growth factor receptor 2 (HER2) expression [1]. This subtype accounts for over 25% of breast cancer-related morbidity. It has a poor prognosis, with its five-year overall survival rate remaining low, despite various treatment regimens [2]. The biological and clinical complexities of TNBC contribute to its aggressive behavior, posing a significant unmet clinical challenge [3]. Current prognostic methods for TNBC rely largely on traditional clinicopathological features and molecular markers [4]. However, these approaches can be costly, invasive, and prone to false positives [5]. Therefore, identifying TNBC-associated prognostic biomarkers and exploring their underlying mechanisms are crucial for improving the prognosis and therapy.

While current studies mainly focus on individual miRNAs or mRNAs as risk markers in cancers [6,7], the prognostic value of hidden regulatory correlations remains largely unexplored. MicroRNAs (miRNAs) are small, non-coding RNA molecules that regulate gene expression by binding to complementary sequences in target mRNAs [8]. The interaction between miRNAs and their target genes has become an important area of research, particularly for drug discovery, as it offers the potential to regulate gene expression networks [9]. miRNAs control approximately one-third of protein-coding genes, highlighting the importance of identifying miRNA–target interactions (MTIs) to better understand disease progression [10]. Moreover, the dynamic network between miRNAs and their target genes can enhance the sensitivity and robustness of associated models [11].

Recent studies have highlighted the role of specific MTIs in TNBC progression and response to treatment. For example, the HIF1A/miR-326/*ITGA5* axis influences chemotherapy response, with lower miR-326 levels correlating with worse survival outcomes [12]. Additionally, subtype-specific interactions, such as the regulation of *INPP4B* by miR-17-92, point to potential therapeutic targets [13]. As shown in Table 1, miRTarBase currently records 12 key MTI pairs involving 11 miRNAs in TNBC [14]. However, these findings have not yet been integrated into comprehensive prognostic models for better patient outcome prediction. Beyond miRNA regulation, gene expression is also controlled by transcription factors (TFs) at the transcriptional level [15]. TFs and miRNAs form intricate regulatory networks, often organized into motifs such as feed-forward loops (FFLs), where two regulators control each other and jointly regulate target genes [16]. These network motifs increase the stability of gene regulation and have proven useful in understanding tumor pathogenesis in various cancers, including prostate cancer, glioblastoma [17], and ovarian cancer [18].

**Figure 1:**
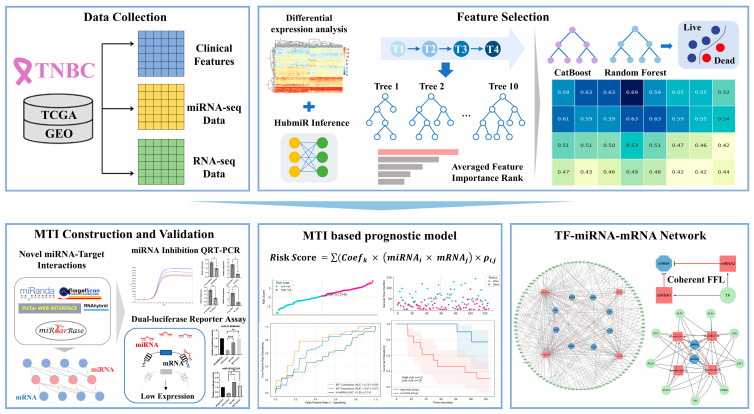
The flowchart of the study. * for *p*-value < 0.05, ** for *p*-value < 0.001, **** for *p*-value < 0.00001.

In this study, we developed a novel MTI-based prognostic model for TNBC in which eight important miRNAs are discovered by differential expression analysis, miRNA–mRNA regulatory correlations inference, and machine learning. By combining bioinformatics analysis with experimental validation, we identified 10 key MTI pairs as prognostic signatures, providing clinical insights for patient risk stratification. This approach uncovers hidden regulatory correlations of TNBC, improving prognostic accuracy and offering potential therapeutic insights. Furthermore, by analyzing feed-forward regulatory loops and network motifs, we provide a comprehensive TF–miRNA–mRNA regulatory landscape of TNBC, expanding our understanding of its regulatory mechanisms and guiding the development of more effective therapeutic strategies. The flowchart of this work is shown in Figure 1.

## 2. Results

### 2.1. Identification of Differentially Expressed miRNAs and mRNAs

**Figure 2:**
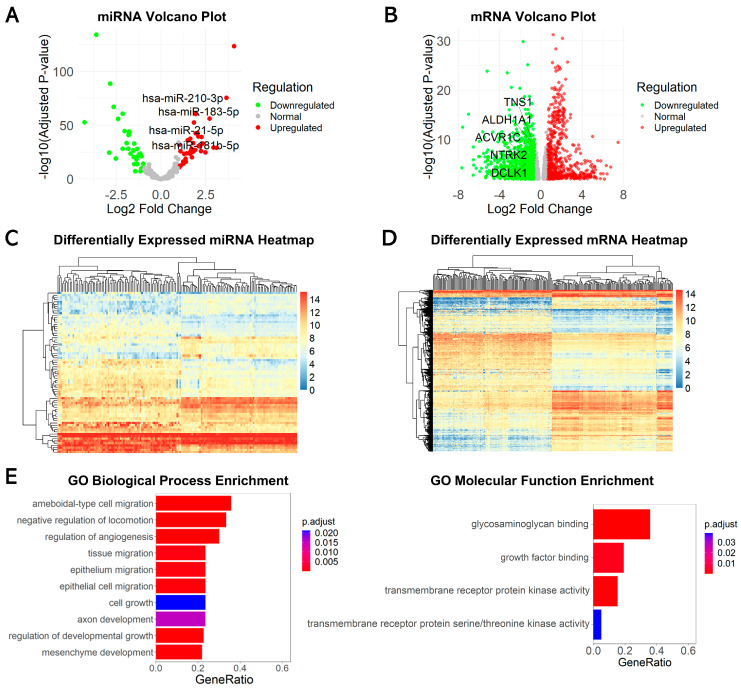
Identification of DEmiRs and DEGs. (**A**) Volcano plot of miRNA differential expression in TNBC samples; (**B**) volcano plot of mRNA differential expression in TNBC samples; (**C**) heatmap of miRNA differential expression between TNBC samples and normal samples; (**D**) heatmap of mRNA differential expression between TNBC samples and normal samples; (**E**) GO functional enrichment for differentially expressed mRNAs. Left: bar chart of top 10 GO biological processes analysis; right: bar chart of GO molecular functions analysis.

The Cancer Genome Atlas (TCGA) dataset analyzed in this study included 126 TNBC samples and 114 adjacent normal breast tissue samples, for which both small RNA and gene expression profiles were available. Heatmaps were used to visualize the expression profiles of miRNAs and mRNAs (Figure 2A,B). A total of 38 upregulated and 34 downregulated miRNAs were identified as significantly differentially expressed (Supplementary Table S2). Additionally, we found 737 upregulated and 796 downregulated mRNAs with significant expression differences (Figure 2C,D).

To understand the biological functions associated with the differentially expressed mRNAs (DEG), we performed Gene Ontology (GO) analysis. The results revealed that the DEGs in TNBC are predominantly involved in processes related to cancer metastasis, such as “tissue migration”, “epithelium migration”, and “regulation of angiogenesis” (Figure 2E). Four molecular functions were notably enriched, including “glycosaminoglycan binding”, “growth factor binding”, and “transmembrane receptor protein kinase activity”. These findings suggest that the DEGs play important roles in cancer cell migration and tumor progression.

To screen miRNAs with strong regulatory capability in TNBC, we used HubmiR [19] to analyze the expression patterns and identify strong regulatory connections. By integrating the paired expression profiles from the TCGA dataset, we identified 168 miRNAs with a contrast value ≥ 0.7, indicating strong regulatory potential. These miRNAs are closely linked to mRNA expression and likely play significant roles in TNBC progression, making them candidates for further analysis.

### 2.2. Screening of Prognostic-Related Hub miRNAs in TNBC

**Figure 3:**
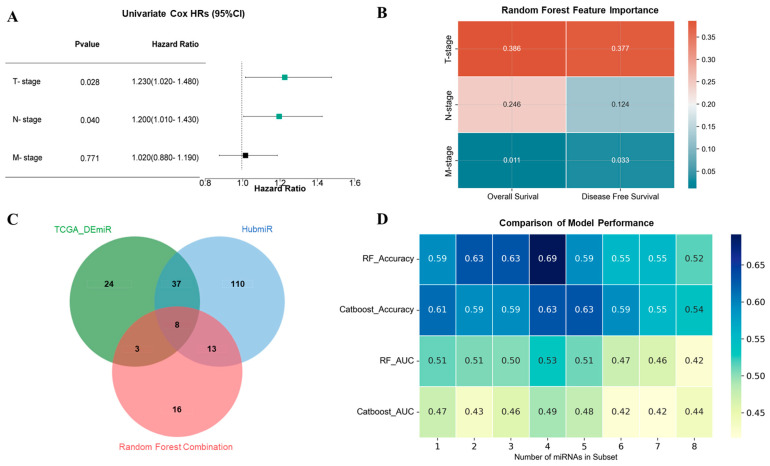
Identification of prognosis-related miRNAs in TNBC. (**A**) A univariate Cox regression analysis was performed to identify key clinical features that affected survival outcomes, the green squares represent significant clinical features, while the black square indicates non-significant features; (**B**) a random forest algorithm was performed to identify key clinical features associated with survival outcomes; (**C**) Venn plot showing TCGA-, HubmiR-, and random forest combination-selected miRNAs; (**D**) performance comparison of different miRNA subsets in terms of median accuracy and median AUC among two machine-learning algorithms.

To identify features most associated with TNBC patient survival, we performed univariate Cox regression analysis. This revealed that both tumor stage (T-stage) and node stage (N-stage) were significantly correlated with survival outcomes, with hazard ratios (HR) of 1.230 (95% CI: 1.020–1.480, *p* = 0.028) for T-stage and 1.200 (95% CI: 1.010–1.430, *p* = 0.040) for N-stage. In contrast, the metastasis stage (M-stage) did not show a significant correlation with survival (HR = 1.020, 95% CI: 0.880–1.190, *p* = 0.771) (Figure 3A). To further assess the importance of these clinical features, we conducted random forest analysis, which confirmed that T-stage had the strongest association with both overall survival (importance score = 0.386) and disease-free survival (importance score = 0.377). N-stage showed moderate importance, with scores of 0.246 for overall survival and 0.124 for disease-free survival, while M-stage had minimal impact (importance scores of 0.011 and 0.033, respectively) (Figure 3B). Based on these findings, we used random forest combination to analyze the TCGA_TNBC dataset and identified 40 miRNAs as key features for T-stage classification.

By integrating three approaches, i.e., differentially expressed miRNAs from the TCGA dataset, miRNA–mRNA regulatory correlations inference by HubmiR (See Section 4), and random forest combination, we identified a core set of hub miRNAs. Eight miRNAs were found to overlap across all three methods (Figure 3C). Given the imbalanced distribution of the survival data, we applied both random forest and CatBoost algorithms to identify the optimal prognostic miRNA subset. Performance metrics indicated that models using four miRNAs (hsa-miR-181b-5p, hsa-miR-21-5p, hsa-miR-210-3p, and hsa-miR-183-5p) provided the best prognostic results, with a significant association with TNBC overall survival (Figure 3D, Supplementary Figure S1).

### 2.3. Identification of Putative miRNA–Target Interaction Network in TNBC

Potential target genes for the selected miRNAs were identified using multiple prediction algorithms, alongside experimentally validated miRNA targets retrieved from miRTarBase. This analysis revealed 179 targets for hsa-miR-210-3p, 188 targets for hsa-miR-21-5p, 337 targets for hsa-miR-181b-5p, and 257 targets for hsa-miR-183-5p. Among these, six targets for hsa-miR-210-3p, 32 targets for hsa-miR-21-5p, 11 targets for hsa-miR-181b-5p, and 15 targets for hsa-miR-183-5p were confirmed in the miRTarBase database (Supplementary Table S3).

**Figure 4:**
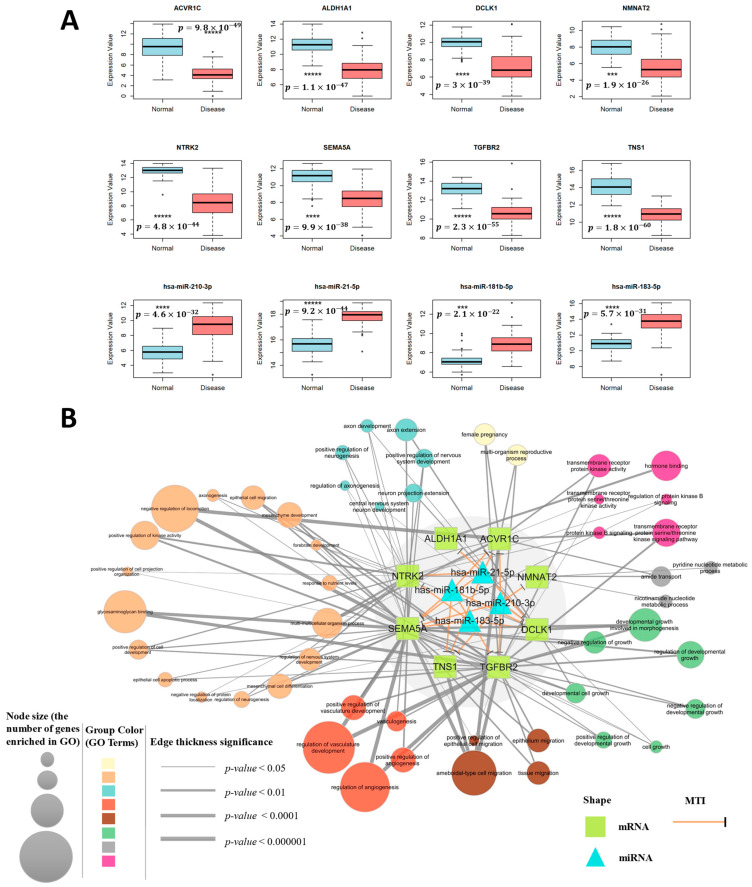
(**A**) Expression profile of selected miRNAs and mRNAs in TNBC data. The box plots show the expression values for normal versus disease samples. Statistical significance is indicated as follows: *** for *p*-value < 0.0001, **** for *p*-value < 0.00001, and ***** for *p*-value < 0.000001; (**B**) the miRNA–mRNA regulatory network in TNBC and a network diagram of individual target gene functions.

Functional enrichment analysis revealed that the DEGs in TNBC were significantly associated with migration-related biological processes. Consistent with previous research, metastasis emerged as a key contributor to poor prognosis in TNBC, given its high invasiveness and early dissemination [20]. Cell migration, in particular, was identified as a critical driver of metastasis [21]. To uncover novel MTIs relevant to TNBC progression, we focused on the common targets of the selected miRNA biomarkers, prioritizing migration-associated genes. As a result, we selected high-confidence predictive targets, particularly those involved in cell migration, and identified eight potential tumor suppressor genes, *TNS1*, *DCLK1*, *SEMA5A*, *ACVR1C*, *NTRK2*, *TGFBR2*, *ALDH1A1*, and *NMNAT2*, as promising candidates for further investigation (Supplementary Tables S4 and S5). The expression levels of these miRNAs and mRNAs in the TNBC dataset are displayed in Figure 4A. Putative miRNA binding sequences in the 3′ UTRs of the target genes are provided in Supplementary Tables S6–S9.

An miRNA–target gene regulatory network was constructed, shown in the gray region of Figure 4B. This network identified 19 predictive interactions between the upregulated miRNAs (hsa-miR-210-3p, hsa-miR-183-5p, hsa-miR-21-5p, hsa-miR-181b-5p) and eight downregulated mRNAs (*TNS1*, *DCLK1*, *SEMA5A*, *ACVR1C*, *NTRK2*, *TGFBR2*, *ALDH1A1*, *NMNAT2*).

To explore the biological mechanisms underlying this miRNA–target gene network, the functional annotation of the target genes is visualized in Figure 4B. The node sizes in the network reflect the number of genes enriched in each pathway, while the colors indicate the similarity of the GO pathways. The thickness of the connecting lines corresponds to the significance of gene enrichment. The clustered functional enrichment analysis revealed that the target genes of the selected miRNAs were primarily enriched in processes related to proliferation, migration, invasion, and apoptosis. Among the eight target genes, *SEMA5A*, *TNS1*, and *TGFBR2* were linked to the cell migration and angiogenesis pathways, while *NTRK2*, *ACVR1C*, and *ALDH1A1* were mainly associated with cell differentiation regulation. *DCLK1* and *NMNAT2* appeared to play roles in abnormal cell growth regulation.

### 2.4. Validation of MTI Pairs

**Figure 5:**
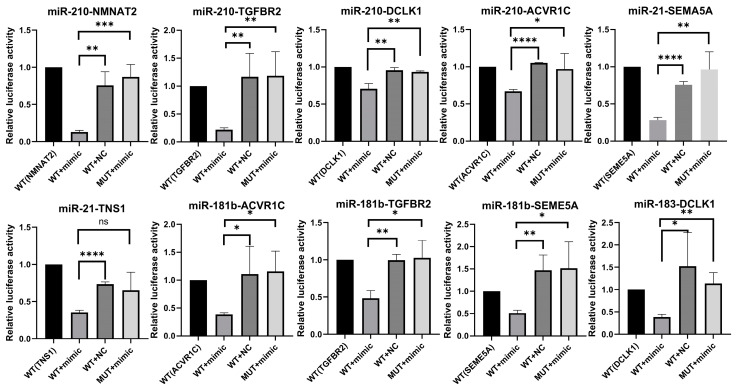
Luciferase reporter assay validation of identified MTI pairs. The bar graphs represent relative luciferase activity in different experimental conditions. Statistical significance is indicated as follows: * for *p*-value < 0.05, ** for *p*-value < 0.001, *** for *p*-value < 0.0001, and **** for *p*-value < 0.00001.

To validate the direct interactions between the selected miRNAs and their target mRNAs, we performed dual-luciferase reporter assays for 19 miRNA–target pairs. Eighteen of these pairs were confirmed, with miRNA mimics significantly inhibiting the expression of their target mRNAs (*p*-value < 0.05) (Supplementary Figure S2). Notably, ten MTIs—miR-210-3p/*NMNAT2*, miR-210-3p/*TGFBR2*, miR-210-3p/*DCLK1*, miR-210-3p/*ACVR1C*, miR-21-5p/*TNS1*, miR-21-5p/*SEMA5A*, miR-181b-5p/*ACVR1C*, miR-181b-5p/*TGFBR2*, miR-181b-5p/*SEMA5A*, and miR-183-5p/*DCLK1*—showed strong interactions between the miRNAs and their target mRNAs. For these pairs, co-transfection with miRNA mimics significantly reduced the luciferase activity of plasmids containing wild-type 3′ UTR fragments of the target genes. At the same time, no effect was observed with the mutated fragments (Figure 5). These results validate the computational predictions of interaction and mRNA binding sites.

### 2.5. miRNA Inhibition Analysis

**Figure 6:**
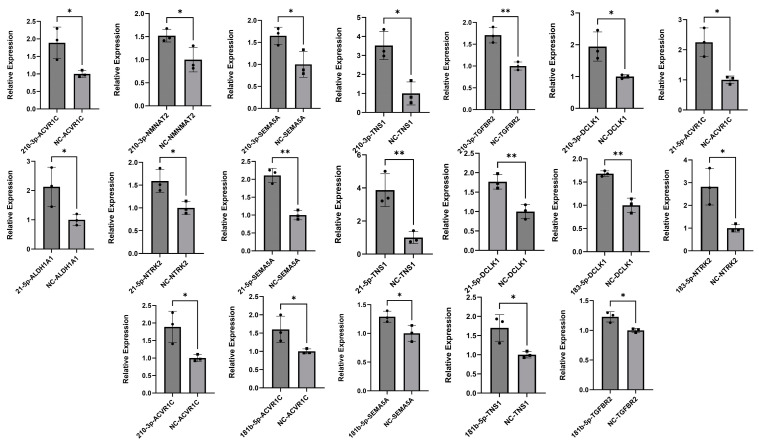
Relative expression of target genes in MDA-MB-231 cells following miRNA inhibitor transfection. Bar graphs represent the relative expression levels of target genes in cells transfected with miRNA inhibitors compared to negative control. Statistical significance is indicated as follows: * for *p*-value < 0.05 and ** for *p*-value *<* 0.001.

To further validate the regulatory impact of these miRNAs, we performed a quantitative real-time PCR (qRT-PCR) assay to assess the effects of miRNA inhibition on target gene expression in MDA-MB-231 cells. The inhibition of miR-210-3p, miR-21-5p, miR-181b-5p, and miR-183-5p led to a significant upregulation of their respective target genes compared to the results for the negative control group (Figure 6). These changes were statistically significant (*p*-value < 0.05), highlighting the robust regulatory role of miRNA inhibition in gene expression.

### 2.6. Identification of Prognosis-Related MTI Risk Model in TNBC

**Figure 7:**
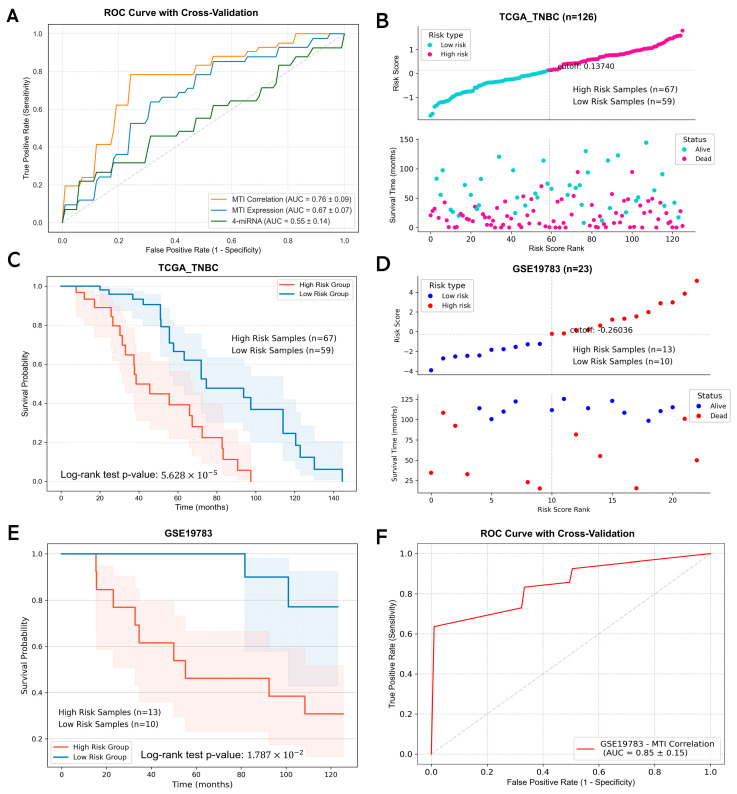
Performance evaluation and validation of the MTI-based prognostic model in TNBC. (**A**) ROC curves comparing the predictive performance of three approaches: 10 MTI correlations, 10 MTI associated gene expression, and 4-miRNA signatures; (**B**) distribution of risk score and overall survival status of the patients based on the risk score in TCGA cohorts. The vertical gray dashed line on the x-axis denotes the risk score cutoff value, and the horizontal gray dashed line on the y-axis indicates the corresponding rank value of the cutoff; (**C**) Kaplan–Meier survival curves for high- and low-risk groups in the TCGA_TNBC cohort; (**D**) distribution of risk score and overall survival status of the patients based on the risk score in GSE19783 cohorts. The vertical gray dashed line on the x-axis denotes the risk score cutoff value, and the horizontal gray dashed line on the y-axis indicates the corresponding rank value of the cutoff; (**E**) Kaplan–Meier survival curves for high- and low-risk groups in the GSE19783 validation cohort; (**F**) ROC curve of the 10 MTI correlation-based model in the GSE19783 dataset.

Based on the top 10 significantly interacted MTI pairs validated by a dual luciferase reporter assay, we utilized Cox regression to construct an MTI-related prognostic model. This model was developed using the TCGA TNBC cohort as the training dataset and the GSE19783 dataset for independent validation. The risk score (RS) for each patient was calculated as a weighted sum of the expression levels of the miRNAs, their target genes, and the Spearman correlation of the miRNA–target pairs. The formula for the risk score is as follows: RS = (−0.139 × expression of miR-183-5p × expression of *DCLK1* × Spearman correlation of miR-183-5p/*DCLK1*) + (−0.032 × expression of miR-210-3p × expression of *DCLK1* × Spearman correlation of miR-210-3p/*DCLK1*) + (0.251 × expression of miR-210-3p × expression of *ACVR1C* × Spearman correlation of miR-210-3p/*ACVR1C*) + (0.036 × expression of miR-210-3p × expression of *TGFBR2* × Spearman correlation of miR-210-3p/*TGFBR2*) + (0.256 × expression of miR-210-3p × expression of *NMNAT2* × Spearman correlation of miR-210-3p/*NMNAT2*) + (−0.240 × expression of miR-21-5p × expression of *SEMA5A* × Spearman correlation of miR-21-5p/*SEMA5A*) + (−0.099 × expression of miR-21-5p × expression of *TNS1* × Spearman correlation of miR-21-5p/*TNS1*) + (0.282 × expression of miR-181b-5p × expression of *ACVR1C* × Spearman correlation of miR-181b-5p/*ACVR1C*) + (0.494 × expression of miR-181b-5p × expression of *SEMA5A* × Spearman correlation of miR-181b-5p/*SEMA5A*) + (0.052 × expression of miR-181b-5p × expression of *TGFBR2* × Spearman correlation of miR-181b-5p/*TGFBR2*). The model’s predictive performance was evaluated using ROC curve analysis, which revealed an area under the curve (AUC) of 0.76 ± 0.09 for the 10 MTI signature. This was superior to the results for models based on miRNA and target expression alone (AUC = 0.67 ± 0.07) or the 4-miRNA signature (AUC = 0.55 ± 0.14) (Figure 7A).

In the TCGA cohort (n = 126), patients were divided into high-risk (67 patients) and low-risk (59 patients) groups, using an optimal cutoff value of 0.137 derived from K-means clustering based on their risk scores (Figure 7B). The Kaplan–Meier survival analysis results showed a significant difference in OS between the groups (log-rank test *p*-value = 5.628 × 10^−5^), with the high-risk group exhibiting significantly poorer survival (Figure 7C).

To validate the robustness of this MTI-based prognostic model, we applied it to the independent GSE19783 dataset. Using a cutoff value of −0.260, we classified 23 patients into high-risk (n = 13) and low-risk (n = 10) groups (Figure 7D). Survival analysis of the validation cohort confirmed the model’s prognostic value, with the high-risk group showing significantly worse disease-free survival outcomes (log-rank test *p*-value = 1.787 × 10^−2^) (Figure 7E). The model also demonstrated strong predictive performance in the validation dataset, with an AUC of 0.85 ± 0.12 (Figure 7F).

In conclusion, the 10 MTI signature provides a robust and reproducible method for risk stratification in TNBC patients, demonstrating its potential for clinical application in prognosis prediction. Compared to previous studies utilizing single feature types, our model shows superior and consistent performance across both training and validation datasets, highlights the utility of the combination of miRNA–mRNA correlation in prognosis prediction, and confirms the robustness and generalizability of our model (Supplementary Table S10). Further survival analysis of the four miRNAs and eight target genes revealed that a lower expression of *ALDH1A1*, *TGFBR2*, *DCLK1*, and *NMNAT2* was associated with worse overall survival. Additionally, a higher expression of hsa-miR-21 and hsa-miR-183 correlated with poorer survival outcomes (Supplementary Figure S3).

### 2.7. Interactions of TFs with miRNAs and mRNAs in TNBC Progression

**Figure 8:**
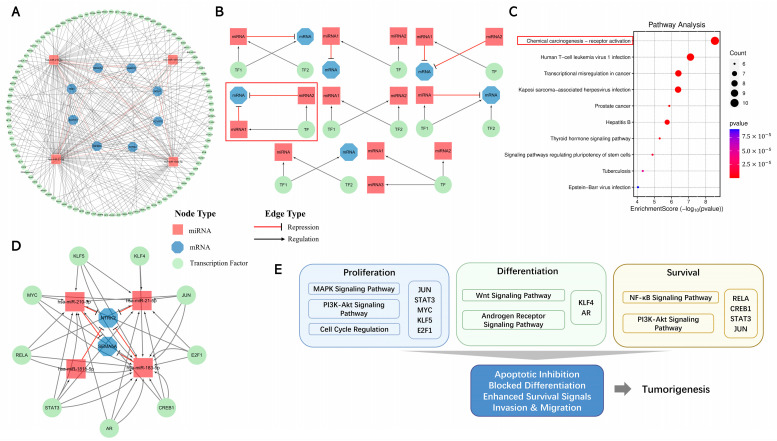
The TF–miRNA–mRNA regulatory network of TNBC. (**A**) A global network of TF–miRNA–mRNA interactions. The edge types are represented as follows: a red line with an inhibition arrow indicates that the miRNA represses the target mRNA, while a gray line with an arrow represents TF regulation on genes, without a specific indication of activation or repression. The nodes are classified into three types: red for miRNAs, blue for mRNAs, and green for TFs; (**B**) eight different types of four-node regulatory motifs. Red circles the motif with coherent FFL; (**C**) KEGG analysis of TFs belonging to coherent FFL. Red circles the most enriched KEGG pathway; (**D**) the coherent FFL motif subnetwork of the most enriched KEGG pathway; (**E**) the functional annotation of TFs in the most enriched KEGG pathway.

By analyzing the transcriptional regulatory relationships (TF–miRNA, TF–mRNA), we constructed a TF–miRNA–mRNA regulatory network (Figure 8A). This network comprised 101 TFs, with 79 regulating miRNAs and 22 influencing mRNAs. To uncover critical regulatory substructures, we used the FANMOD tool to identify four-node motifs based on the interactions between miRNAs, mRNAs, and TFs. Eight distinct regulatory motifs were identified, each meeting the criteria of a z-score greater than two and a *p*-value less than 0.05 (Figure 8B). Among these motifs, we focused on those involving coherent feed-forward loops (FFLs). A total of 71 FFLs were identified, involving 45 TFs, four miRNAs, and two mRNAs (Supplementary Table S11).

To further investigate the functional significance of these FFLs, we conducted a Kyoto Encyclopedia of Genes and Genomes (KEGG) pathway analysis, which revealed 66 significant pathways (*p*-value < 0.05) (Supplementary Table S12). The top 10 pathways are presented in Figure 8C, with a particular emphasis on the most significant pathway, hsa05207: chemical carcinogenesis-receptor activation, which involved 10 TFs. The pathway related to transcriptional misregulation in cancer was also enriched. Collectively, we constructed a functionally associated regulatory network significantly linked to cell proliferation and metastasis in TNBC, as depicted in Figure 8D.

This network comprises 18 coherent FFLs, with TFs involved in key pathways such as the PI3K-Akt signaling pathway, MAPK signaling pathway, Wnt signaling pathway, androgen receptor signaling pathway, NF-κB signaling pathway, and cell cycle regulation in cancers, stepwisely contributing to dysregulation in cell proliferation, differentiation, and survival. The abnormalities in these processes are major drivers of tumorigenesis. This network highlights a precise and rapid regulatory mechanism that enables cells to swiftly adjust their proliferation, invasion, and metastasis capabilities in response to changes in the tumor microenvironment. The nodes in this network may serve as potential TNBC diagnostic biomarkers.

## 3. Discussion

In this study, we introduced a prognostic model that integrates novel miRNA–mRNA interactions to elucidate the complex molecular mechanisms driving TNBC progression and outcomes. By focusing on miRNA-mediated regulatory networks and associated expression profiles, we aimed to uncover the intricate interactions underlying TNBC’s aggressive behavior and poor prognosis. To address this, we identified key miRNAs linked to mRNA expression and tumor classification, highlighting significant miRNA–target interactions through experimental validation. The proposed prognostic risk model successfully stratified TNBC patients by survival outcomes across both training and validation cohorts, showcasing its ability to capture the complex regulatory landscape beyond traditional single-marker approaches.

To systematically identify candidate biomarkers, we employed differential expression analysis, HubmiR neural network analysis, and tumor-stage classification, resulting in the identification of four key miRNAs (hsa-miR-181b-5p, hsa-miR-21-5p, hsa-miR-210-3p, and hsa-miR-183-5p) significantly associated with survival outcomes. Functional enrichment analysis suggests that these miRNAs play pivotal roles in TNBC pathology. For instance, hsa-miR-210-3p is linked to lymph node metastasis and trastuzumab sensitivity [22,23], while hsa-miR-21-5p promotes tumor invasion, metastasis, and therapy resistance [24]. Additionally, hsa-miR-181b-5p was identified to enhance inflammation and cancer cell migration [25], and hsa-miR-183-5p exacerbates breast cancer development, promoting tumor differentiation, invasion, and metastasis [26]. These miRNAs hold potential as biomarkers, advancing our understanding of TNBC diagnosis, treatment, and prognosis. Furthermore, based on identified hub miRNAs, eight downregulated mRNA targets with novel interaction relationships were validated through dual-luciferase reporter assay and miRNA-inhibition analysis. Functional enrichment analysis showed that these target mRNAs may be involved in migration, metastasis, and the cell cycle in TNBC, all of which are highly relevant to cancer cell behavior.

Considering the prognosis risk model, previous approaches typically focused on identifying isolated miRNA [27,28], mRNA [29], or TF [30] biomarkers and their respective applications. In contrast, our MTI-based model integrates both miRNA and mRNA expression profiles, utilizing their correlations as model features for more comprehensive and accurate prognosis in TNBC. Advanced machine learning techniques, including random forest and CatBoost, enhanced feature selection, model interpretability, and accuracy, while minimizing overfitting. Additionally, the 10 experimentally validated MTI signatures provide biological relevance and strengthen the model’s robustness. Cross-cohort validation demonstrated superior predictive performance compared to that of previous methods, successfully stratifying patients into high-risk and low-risk groups in both the TCGA and independent GEO datasets. High-risk patients based on these MTI signatures were strongly associated with poorer overall and disease-free survival, further emphasizing the prognostic power of our network-based approach and underscoring the potential clinical utility of our model in improving TNBC prognosis prediction.

Further investigation of TF–miRNA–mRNA interactions revealed key regulatory mechanisms through the identification of feed-forward loops (FFLs). Using FANMOD for motif detection, we uncovered 71 coherent FFLs involving 45 transcription factors (TFs), four miRNAs, and two mRNAs. These loops highlight critical regulatory nodes where pathways converge to influence cellular processes such as proliferation, invasion, and metastasis. Pathway enrichment analyses pinpointed the MAPK and PI3K-Akt signaling pathways and cell cycle regulation as central drivers of TNBC’s aggressive phenotype. The involvement of nine TFs in these pathways underscores their potential as therapeutic targets. Notably, hsa-miR-21 and hsa-miR-183, heavily embedded in these FFLs, emerged as significant markers for survival, making them compelling candidates for future therapeutic interventions.

In summary, this study presents a novel MTI-based prognostic model that combines regulatory network information to improve prognostic prediction in TNBC. By incorporating miRNA–target interactions, we demonstrated improved accuracy and robustness compared to those of traditional single-marker methods. Additionally, the analysis of TF–miRNA–mRNA regulatory networks provided deeper insights into TNBC progression, uncovering potential biomarkers and therapeutic targets. Nevertheless, as a retrospective bioinformatics study, our findings require validation through multicenter prospective studies to confirm their clinical relevance and identify independent prognostic factors. Future research will expand this network-based approach to other cancer types and integrate additional regulatory layers, such as epigenetic modifications and protein–protein interactions, to further improve prognostic accuracy and uncover novel therapeutic opportunities.

## 4. Materials and Methods

### 4.1. Data Collection

Gene and miRNA expression profiles, as well as the clinical data of patients with triple-negative breast cancer, were collected from two public datasets: TCGA_BRCA data from The Cancer Genome Atlas (TCGA) [31] and GSE19783 data from Gene Expression Omnibus (GEO, http://www.ncbi.nlm.nih.gov/geo/, accessed on 24 January 2024). These datasets encompass the expression profile of tumor tissues and adjacent normal tissues from breast cancer patients across subtypes. According to the clinical immunohistochemistry results, samples from TCGA and GEO, with negative status for ER, HER2, and PR, were selected as TNBC samples for further study [32]. Specifically, the TCGA dataset included expression data for 20,530 mRNAs and 1882 miRNAs, while the GSE19783 dataset contained 29,746 mRNAs and 881 miRNAs.

### 4.2. Differential Expression Analysis of miRNAs and mRNAs

DESeq2 was used to analyze the differential expression of RNA-Seq data and miRNA-Seq data from the TCGA_TNBC dataset [33]. The raw expression data for both mRNAs and miRNAs were normalized using the DESeq2 default method, which adjusts for library size differences and stabilizes variance. To filter out low-expression genes and miRNAs, thresholds were applied based on the DESeq2-processed results. To capture genes and miRNAs with modest but potentially biologically relevant expression changes, we applied a slightly less stringent threshold of |log2FC| ≥ 1 in the initial screening. This was combined with stringent statistical significance (FDR < 0.01) and averaged expression level filters to balance sensitivity and specificity. Differentially expressed miRNAs (DEmiR) were selected based on the following criteria: Benjamini–Hochberg adjusted *p*-value < 0.01 with average | log2FC| ≥ 1 and baseMean (the average of gene expression across all samples) ≥ 50. Differentially expressed mRNAs were selected based on adjusted *p*-value < 0.01, an average | log2FC | ≥ 1, and baseMean (the average gene expression across all samples) ≥ 100.

### 4.3. Learning miRNA and mRNA Regulatory Correlation via a Neural Network

The expression patterns of miRNA can be learned from the expression level of mRNA, if a tight regulation between the miRNAs and genes exists. A method based on a four-layer neural network, called *HubmiR* [19], was previously developed by our team to learn the expression pattern of miRNAs from mRNA in terms of pairing expression profiles retrieved from the TCGA database [31] among 33 types of cancer. It was proven to be robust and generalizable on both bulk and single-cell sequencing datasets. In this study, mRNA expression profiles were z-score normalized before being input into the HubmiR tool, which generated inferred relative miRNA expression levels. To identify key regulatory miRNAs in TNBC, we calculated the contrast value, defined as the absolute difference between inferred miRNA expression under TNBC and normal conditions. miRNAs with a contrast value ≥ 0.7 were selected for further analysis.

### 4.4. Selection of the Prognosis-Related miRNAs

First, the tumor stage (T-stage), metastasis stage (M-stage), node stage (N-stage), survival time, and event of the TNBC sample were analyzed using a univariate Cox regression and random forest algorithm to identify which feature is most associated with the TNBC patients’ survival outcome. By applying random forest combination, miRNAs above the 85th percentile were selected as key features for T-stage classification. Intersections of selected miRNAs across the TCGA datasets, HubmiR, and the random forest combination were regarded as hub miRNAs of TNBC. To determine the superior set of miRNAs for prognostic prediction, random forest and CatBoost algorithms were applied.

### 4.5. miRNA Target Gene Identification

Four upregulated miRNAs (hsa-miR-210-3p, hsa-miR-183-5p, hsa-miR-21-5p, hsa-miR-181b-5p) were subjected to target prediction. The mRNA targets of these four up-regulated miRNAs from TCGA were predicted using four in silico miRNA target prediction tools (TargetScan [34], MiRanda [35], RNAhybrid [36], Pictar [37]). Experimentally validated MTI pairs were curated using the miRTarBase [14]. To further screen potential direct targets, various criteria were employed. Specifically, a MiRanda score of ≥140, a MiRanda minimum free energy (MFE) of ≤−10, and a TargetScan seed type of ≥7mer-A1 [38] for the predicted targets were considered favorable choices. Using miRTarBase [14], experimentally validated miRNA–target gene interaction pairs were curated, excluding those that have already been experimentally confirmed. The remaining miRNA–target gene interaction pairs were selected for further analysis.

### 4.6. Cell Culture

The MDA-MB-231 breast cancer cell line was obtained from the American Type Culture Collection (ATCC) [39] and cultured in high-glucose Leibovitz’s L-15 Medium, supplemented with 10% fetal bovine serum (FBS) and 1% penicillin/streptomycin. The cells were maintained in a humidified incubator at 37 °C without CO_2_.

### 4.7. Cell Transfection

The direct interaction between miRNA and the target genes was validated using the dual-luciferase reporter assay [40]. To perform this assay, 3′-UTR reporter harboring predicted binding sites for hsa-miR-210-3p, hsa-miR-21-5p, hsa-miR-183-5p, and hsa-miR-181b-5p were cloned onto a pmirGLO vector, and the sequences of the constructed plasmid were verified by sequencing (GENERAL BIOL, Chuzhou, China). The transfection experiments unfolded when the cellular density reached the optimal range of 70–90% after being seeded into a 24-well plate using an antibiotic-free medium to minimize potential confounding effects. Plasmids constructed with 3′UTR of genes, followed by the employment of a dual-luciferase expressing open reading frame, with or without miRNA mimics or scrambled miRNA (negative control) mimics, were transfected into cells using Lipo2000, followed by 24 h incubation.

### 4.8. Dual-Luciferase Reporter Assay Detection

The transfected cells were subsequently lysed using Passive Lysis 1X Buffer (Promega, Beijing, China). Subsequently, a highly sensitive and precise FlexStation3 fluorescence detector (Promega, Beijing, China) was employed to measure the intensity of firefly luciferase at a wavelength of 570 nm and the intensity of Renilla luciferase at a wavelength of 480 nm. Statistical significance was determined using a one-tailed *t*-test.

### 4.9. Quantitative Real-Time PCR (qRT-PCR)

To identify the effect of miRNA inhibitor transfection on target gene expression, qRT-PCR was performed, aiming to elucidate the potential impact of all miRNA–target interactions on disease pathogenesis. MDA-MB-231 cells were transfected with inhibitors targeting miR-210-3p, miR-21-5p, miR-181b-5p, and miR-183-5p, along with a negative control inhibitor to modulate specific miRNA expression. Following transfection, total RNA was extracted from the cells using TRIzol (Invitrogen cat: 15596026) (Shanghai, China). After concentration and purity measurement, SuperScriptIII Reverse Transcriptase (Invitrogen, Shanghai, China) and miRcute Plus miRNA first-stand cDNA kit (TIANGEN, Beijing, China) were used to generate first-strand cDNA for mRNA. Specific primers (Supplementary Table S1) for target genes of the identified miRNAs, along with GAPDH as a control, were designed. cDNA generated from 1 μg of RNA was subjected to amplification using Applied Biosystems Quant Studio 6.0 (Carlsbad, CA, USA) with a PowerUp SYBR Green Master Mix (Invitrogen, Shanghai, China). This approach integrates miRNA modulation with gene expression analysis, offering insights into miRNA-mediated regulatory mechanisms in disease contexts.

### 4.10. Identification of the MTI Prognostic Risk Model

We evaluated the correlation between mRNA and miRNA expression data using the Spearman correlation coefficient ($\rho_{i,j})$. A prognosis prediction model was developed using the following formula: $\mathrm{risk}\;\mathrm{score}\;\left(\mathrm{RS}\right)=\sum (Coef_{k}\times \left(\exp\left(miRNA_{i}\right)\times \exp\left(mRNA_{j}\right)\right)\times \rho_{i,j}$. In this formula, the expressions of miRNA ($\exp\left(miRNA_{i}\right)$) and mRNA ($\exp\left(mRNA_{j}\right)$) represent their respective contributions to the prognosis of TNBC. The Spearman correlation coefficient ($\rho_{i,j}$) measures the strength of the regulatory relationship between each miRNA and its corresponding mRNA target, capturing biologically relevant interactions. The regression coefficients ($Coef_{k}$), derived from Cox regression analysis, quantify the contribution of each miRNA–mRNA pair to the overall risk score. The prediction ability of the MTI prognostic risk model was assessed by the receiver operating characteristics curve (ROC) and area under the ROC curve (AUC). With the MTI prognostic model, the TNBC patients were stratified into low- and high-risk score (RS) groups based on the cutoff value. To ensure an unbiased selection process, we applied K-means clustering (k = 2) to the risk scores, dividing patients into high-risk and low-risk groups in an unsupervised manner. The resulting cutoff value corresponded to the centroid that best separated these two clusters. This approach ensures an objective classification of risk categories. The correlation between miRNA expression levels and disease-free survival (DFS) and overall survival (OS) was assessed using the Kaplan–Meier method and a log-rank test (Mantel–Cox).

### 4.11. Upstream TFs Identification

Target mRNA and miRNA upstream transcription factors were identified using the TRANSFAC database [41]. Initially, gene names were input, and based on predicted results, with both core similarity score (CSS) and matrix similarity score (MSS) thresholds set at 1, the relationships between TFs and miRNA, as well as TFs and mRNA, were determined. Subsequently, these relationships were visualized using Cytoscape software, version 10.2 [42].

### 4.12. Detection of Four-Node Regulatory Motifs

Given the identified TF–miRNA, miRNA–mRNA, and TF–mRNA interaction pairs, different types of four-node motifs were identified using FANMOD software, (https://www.encodeproject.org/software/fanmod/, accessed on 16 December 2024) [43] in the TF–miRNA–mRNA regulatory networks. Each motif type was assessed for significance through the generation of random networks. A total of 1000 random networks were constructed to serve as a comparison with the original input network. During the network randomization process, a local constant model was applied, where edges with identical relationships were swapped three times. Z-scores were calculated for each motif type, representing the difference between the frequency of motifs in the real network and the mean frequency in the random networks, normalized by the standard deviation. A higher z-score indicates greater motif significance. From the range of motif types identified in the results, those with four-node structures that exhibited z-scores greater than 2.0 and *p*-values less than 0.05 were deemed statistically significant.

### 4.13. Functional Enrichment Analysis

To explore the biological functions associated with the genes, Gene Ontology (GO) enrichment analysis and Kyoto Encyclopedia of Genes and Genomes (KEGG) pathway analysis [44] were conducted. The clusterProfiler [45] package in R was employed for the functional annotation of the target genes and identification of their Gene Ontology (GO) terms within biological processes. Subsequently, GO terms with a Benjamini–Hochberg adjusted *p*-value < 0.05 were selected.

### 4.14. Survival Analysis

Survival analysis was separately performed on candidate miRNAs and target genes using the survminer package in R [46]. MaxStat, version 0.7-25 [47] was employed to calculate the optimal cutoff values for mRNA and miRNA expression levels.

### 4.15. Statistical Analysis

R software (version 4.4.2) and GraphPad Prism version 10 (San Diego, CA, USA) were used to perform all statistical analyses, including graph/plot generation and statistical hypothesis testing. All statistical tests with a *p*-value < 0.05 were considered significant.

## Figures and Tables

**Table 1:** Previously characterized miRNA target interactions in TNBC.

Diseases	miRNAs	Target Genes
Triple-negative breast cancer	hsa-miR-542-3p	*BIRC5*
	hsa-miR-31-5p	*SATB2*
	hsa-miR-218-5p	*SOST, SFRP2*
	hsa-miR-498	*BRCA1*
	hsa-miR-455-3p	*EI24*
	hsa-miR-27a-3p	*GSK3B*
	hsa-miR-155-3p	*NLRP3*
	hsa-miR-496	*Del-1*
	hsa-miR-96-3p	*BRCA1*
	hsa-miR-10b-3p	*BRCA1*
	hsa-miR-199a-5p	*TGFB2*

## Data Availability

The original data presented in the study are openly available in cBioPortal at https://www.cbioportal.org/ and GEO at https://www.ncbi.nlm.nih.gov/geo/, accessed on 24 January 2024.

## Supplementary Materials

The following supporting information can be downloaded at: https://www.mdpi.com/article/10.3390/ijms26051916/s1.
