# Supplementary material for: Unveiling Novel miRNA–mRNA Interactions and Their Prognostic Roles in Triple-Negative Breast Cancer: Insights into miR-210, miR-183, miR-21, and miR-181b
Source: Int J Mol Sci. 2025 Feb 23;26(5):1916. doi: 10.3390/ijms26051916 (PMC11899986; doi:10.3390/ijms26051916)
Supplement: Supplementary file 1 [file ijms-26-01916-s001.zip › TNBC supplementary_250117.pdf]

Supporting Information

Unveiling Novel miRNA-mRNA Interactions and Their Prognostic Roles in Triple-Negative Breast Cancer: Insights into miR-210, miR-183, miR-21 and miR-181b

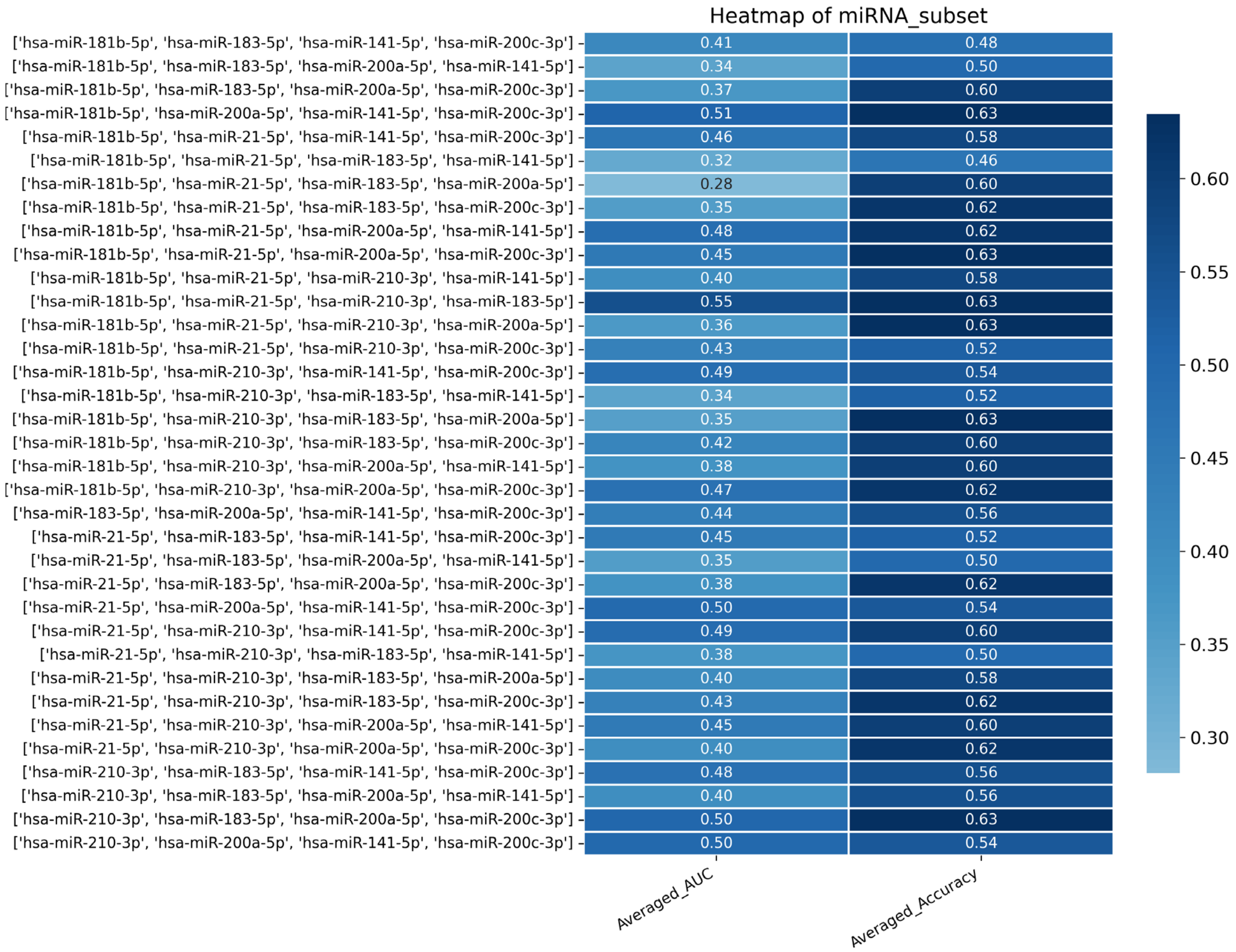

Supplementary Figure S1. This heatmap shows the averaged AUC and accuracy for miRNA subsets. Each row represents a specific combination of miRNAs with miRNA subsets=4.

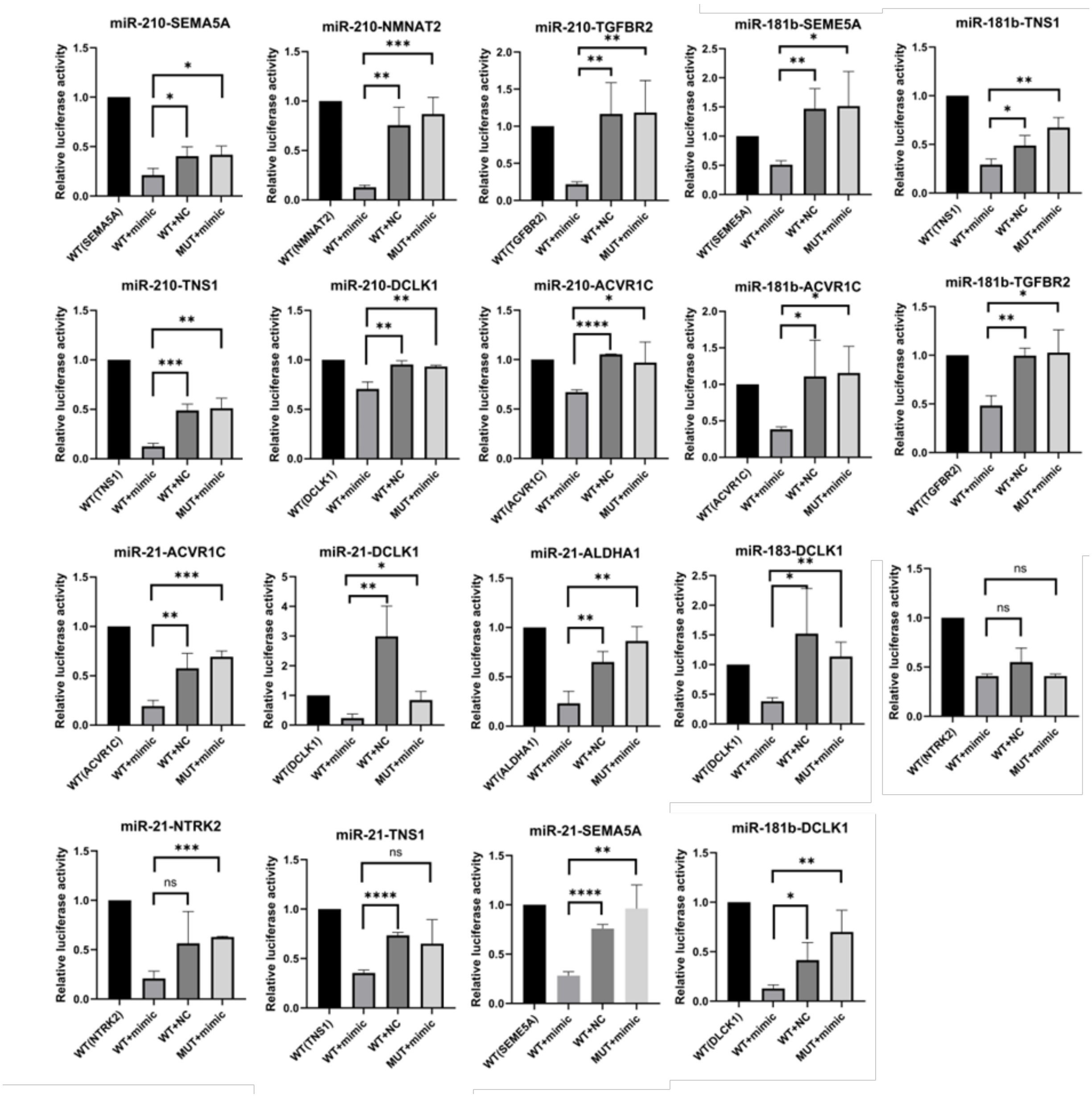

Supplementary Figure S2. A total of 19 validated MTI pairs by dual-luciferase reporter assay. 18 MTI pairs whose miRNA mimics (WT mimic) were validated to significantly (p-value < 0.05) inhibit target mRNAs expression.

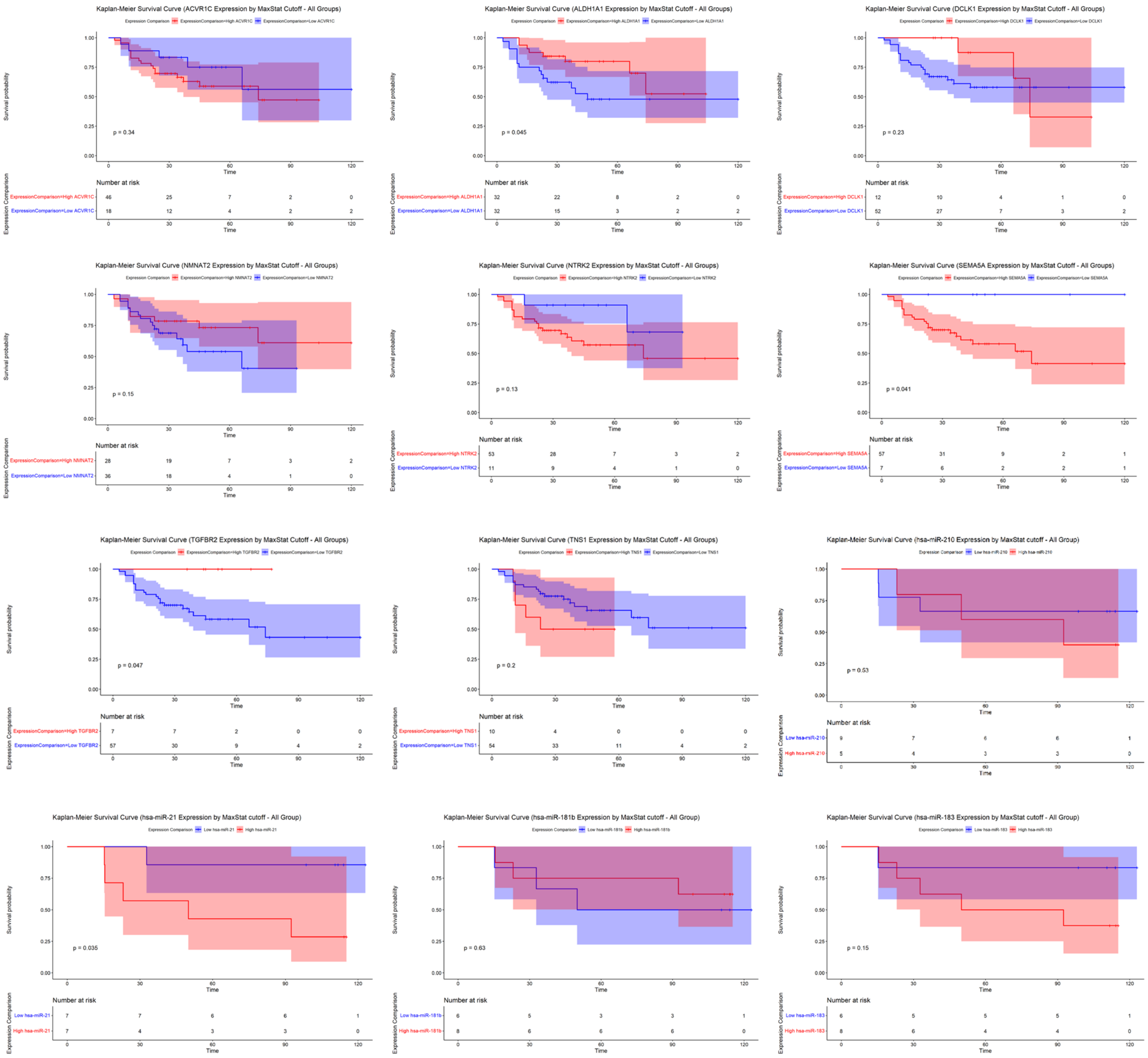

Supplementary Figure S3. Independent survival curves for 8 mRNAs and 4 miRNAs.

Supplementary Table S1. Specific primers sequence for target genes in qRT-PCR.

Primer name	Sequence(5'to3')
SEMA5A-F	GGAACCTGTGTTATAGCATGGC
SEMA5A-R	GCACTGAGTCGTACCCTGG
TGFBR2-F	GTAGCTCTGATGAGTGCAATGAC
TGFBR2-R	CAGATATGGCAACTCCCAGTG
ACVR1C-F	TGGGAACCAAGAGGTATATGGC
ACVR1C-R	GTCAGCTCGTTTGAAGGACTC
DCLK1-F	ACTTCGACGAGCGGGATAAG
DCLK1-R	GGGCCTCAAAAGATCGGAACC
TNS1-F	AGCGGAGACCTGACATCAC
TNS1-R	CGGTTTCCCTTGTTGTGTAGAAC
NTRK2-F	ACCCGAAACAAACTGACGAGT
NTRK2-R	AGCATGTAAATGGATTGCCCA
ALDH1A1-F	CTGCTGGCGACAATGGAGT
ALDH1A1-R	CGCAATGTTTTGATGCAGCCT
NMNAT2-F	ACGGTGATGCGGTATGAAGAG
NMNAT2-R	CACCTCCATATCTGCCTCGTT

Supplementary Table S2A. Characteristics of differentially expressed miRNAs. 38 up-regulated was regarded as significantly differentially expressed.

miRNA Name	baseMean	log2FoldChange	lfcSE	stat	pvalue	padj
hsa-miR-148a-5p	52.08718	1.092794	0.147551	7.406207	1.30E-13	3.43E-13
hsa-miR-181c-3p	59.63931	1.693972	0.149766	11.31076	1.16E-29	7.12E-29
hsa-miR-98-5p	63.04311	1.069451	0.098438	10.8642	1.71E-27	9.08E-27
hsa-miR-203b-3p	67.91374	3.099173	0.269457	11.50154	1.30E-30	8.70E-30
hsa-miR-142-5p	78.84892	2.088753	0.176423	11.83948	2.44E-32	1.76E-31
hsa-miR-99b-3p	87.60559	1.039252	0.141562	7.341311	2.12E-13	5.52E-13
hsa-miR-151a-5p	95.16521	1.00405	0.083364	12.04412	2.08E-33	1.55E-32
hsa-miR-92b-3p	106.3736	1.361367	0.148371	9.175431	4.50E-20	1.65E-19
hsa-miR-128-3p	137.6591	1.472947	0.114686	12.84334	9.37E-38	9.44E-37
hsa-miR-146a-5p	175.0579	1.004597	0.145477	6.905535	5.00E-12	1.21E-11
hsa-miR-200a-3p	185.7795	1.802	0.166958	10.7931	3.71E-27	1.90E-26
hsa-miR-15b-5p	210.8117	1.237318	0.155839	7.939724	2.03E-15	5.83E-15
hsa-miR-181a-3p	253.8288	1.224654	0.143843	8.513828	1.68E-17	5.65E-17
hsa-miR-106b-5p	270.1794	1.916626	0.11462	16.72155	9.13E-63	3.22E-61
hsa-miR-1307-5p	284.3978	4.066965	0.170129	23.90521	2.70E-126	3.81E-124
hsa-miR-196a-5p	331.2507	2.22059	0.205245	10.8192	2.79E-27	1.46E-26
hsa-miR-20a-5p	341.4747	1.639362	0.15228	10.76542	5.01E-27	2.52E-26
hsa-miR-455-3p	351.1708	2.166291	0.184004	11.77304	5.37E-32	3.79E-31
hsa-miR-192-5p	384.3004	1.436461	0.138392	10.37963	3.07E-25	1.42E-24
hsa-miR-17-3p	385.612	2.072982	0.153983	13.46239	2.60E-41	3.19E-40
hsa-miR-181b-5p	417.978	1.736042	0.163448	10.62136	2.37E-26	1.17E-25
hsa-miR-155-5p	469.9399	1.78842	0.144427	12.38287	3.24E-35	2.94E-34
hsa-miR-200a-5p	479.2323	1.890319	0.170675	11.07555	1.65E-28	9.12E-28
hsa-miR-200b-3p	491.5279	1.374092	0.16203	8.480504	2.24E-17	7.44E-17
hsa-miR-210-3p	526.9285	3.636461	0.19454	18.6926	5.69E-78	3.21E-76
hsa-miR-141-5p	595.3175	1.710235	0.178876	9.560984	1.17E-21	4.45E-21
hsa-miR-17-5p	618.0433	2.01176	0.143247	14.04402	8.38E-45	1.18E-43
hsa-miR-141-3p	718.3573	2.243477	0.167702	13.37777	8.16E-41	9.58E-40
hsa-miR-1307-3p	1172.548	1.279161	0.124639	10.26291	1.04E-24	4.42E-24
hsa-miR-21-3p	1808.803	1.925635	0.137927	13.96127	2.69E-44	3.61E-43
hsa-miR-142-3p	2635.024	2.317584	0.189048	12.25924	1.50E-34	1.21E-33
hsa-miR-9-5p	4429.852	2.918719	0.252091	11.57803	5.33E-31	3.66E-30
hsa-miR-93-5p	5649.714	1.619767	0.123689	13.09549	3.49E-39	3.65E-38
hsa-miR-203a-3p	6802.634	2.47099	0.233	10.60509	2.82E-26	1.37E-25
hsa-miR-200c-3p	9179.936	1.263671	0.15339	8.238295	1.75E-16	5.47E-16
hsa-miR-183-5p	10003.58	2.70525	0.167897	16.11256	2.08E-58	5.87E-57
hsa-miR-182-5p	21333.35	1.612019	0.156045	10.33048	5.13E-25	2.30E-24
hsa-miR-21-5p	151602.1	1.826278	0.117439	15.55089	1.57E-54	3.40E-53

Supplementary Supplementary Table S2B. Characteristics of differentially expressed miRNAs. 34 down-regulated was regarded as significantly differentially expressed.

miRNA Name	baseMean	log2FoldChange	lfcSE	stat	pvalue	padj
hsa-miR-381-3p	53.17194	-1.35441	0.140758	-9.62232	6.44E-22	2.52E-21
hsa-let-7b-3p	57.41032	-1.1637	0.112573	-10.3372	4.78E-25	2.17E-24
hsa-let-7c-3p	58.84992	-1.83576	0.161868	-11.3411	8.21E-30	5.14E-29
hsa-miR-145-3p	63.10567	-1.8176	0.132403	-13.7278	6.92E-43	8.87E-42
hsa-miR-675-3p	70.12999	-1.14516	0.205048	-5.58482	2.34E-08	4.46E-08
hsa-miR-328-3p	78.44105	-1.00152	0.12522	-7.99806	1.26E-15	3.71E-15
hsa-miR-195-5p	79.11749	-1.7743	0.125854	-14.0981	3.90E-45	5.79E-44
hsa-miR-30c-2-3p	81.26334	-1.00261	0.161576	-6.20516	5.46E-10	1.15E-09
hsa-miR-337-3p	97.21767	-2.14989	0.189555	-11.3418	8.15E-30	5.14E-29
hsa-miR-378a-5p	165.0257	-1.5439	0.140352	-11.0002	3.81E-28	2.07E-27
hsa-miR-365a-3p	185.7407	-1.93145	0.156235	-12.3624	4.17E-35	3.68E-34
hsa-miR-365b-3p	185.7496	-1.93119	0.156249	-12.3597	4.32E-35	3.69E-34
hsa-miR-139-5p	192.785	-3.61603	0.14496	-24.945	2.42E-137	6.82E-135
hsa-miR-335-3p	192.9582	-1.50708	0.192869	-7.81397	5.54E-15	1.52E-14
hsa-miR-144-5p	228.9101	-2.52207	0.271846	-9.27756	1.73E-20	6.43E-20
hsa-miR-126-5p	351.0346	-1.3379	0.118868	-11.2554	2.18E-29	1.28E-28
hsa-miR-199b-5p	402.5922	-1.79334	0.125933	-14.2404	5.14E-46	8.06E-45
hsa-miR-486-5p	704.7526	-4.27183	0.273643	-15.611	6.13E-55	1.44E-53
hsa-miR-193a-5p	775.4446	-1.49775	0.12208	-12.2686	1.34E-34	1.11E-33
hsa-miR-127-3p	939.0918	-1.08773	0.165457	-6.57406	4.90E-11	1.09E-10
hsa-miR-451a	1142.606	-2.89859	0.275	-10.5403	5.63E-26	2.65E-25
hsa-miR-125a-5p	1172.655	-1.18555	0.115091	-10.301	6.97E-25	3.03E-24
hsa-miR-379-5p	1227.924	-1.37624	0.15235	-9.03339	1.66E-19	5.94E-19
hsa-miR-125b-5p	1991.662	-2.14904	0.128201	-16.763	4.55E-63	1.83E-61
hsa-miR-99a-5p	2099.514	-2.40155	0.149612	-16.0519	5.55E-58	1.42E-56
hsa-miR-126-3p	4269.053	-1.22941	0.120568	-10.1968	2.05E-24	8.62E-24
hsa-miR-145-5p	4370.944	-2.65123	0.150447	-17.6224	1.66E-69	7.80E-68
hsa-let-7c-5p	5673.72	-2.03475	0.142017	-14.3276	1.47E-46	2.59E-45
hsa-miR-205-5p	6192.932	-1.44713	0.258591	-5.59622	2.19E-08	4.20E-08
hsa-miR-100-5p	8656.796	-1.70402	0.165021	-10.3261	5.37E-25	2.37E-24
hsa-miR-30a-3p	18938.34	-1.34543	0.171786	-7.83204	4.80E-15	1.33E-14
hsa-let-7b-5p	29048.65	-1.09384	0.112334	-9.73731	2.09E-22	8.30E-22
hsa-miR-10a-5p	30782.28	-1.53386	0.159503	-9.61649	6.81E-22	2.63E-21
hsa-miR-10b-5p	175692.2	-2.84084	0.140118	-20.2747	2.15E-91	1.52E-89

Supplementary Supplementary Table S3. The potential targets for the selected miRNAs using different tools.

MiRNA	Regulation (miRNAs)	Count of targets	Regulation (mRNAs)	Experimentally validated target mRNAs		Computationally predicted target mRNAs
				Strong evidences	Less strong evidences	
hsa-miR-210-3p	Up	179	Down	HIF3A (MIRT054794), EHD2 (MIRT054209), GPD1L (MIRT003165), FOXN3 (MIRT054204)	RUNX1T1 (MIRT554511), MITF (MIRT473190)	KIAA0408, AQP7P1, TUSC5, GPD1, CD300LG, C14orf180, ACVR1C, KLB, CYP1A1, LIPE, GPAM, ACACB, SCARA5, CRHBP, ALDH1L1, OXTR, CHRDL1, NNAT, CLDN19, MAP1LC3C, SCN2B, GHR, FAM196B, SORBS1, ITIH5, CASQ2, SCN7A, GRIA4, MASP1, MYH11, KCNE1, G0S2, NPR1, PRRT4, C1QTNF7, TNS1, ITGA7, SLC16A7, ADCY5, KLHL31, FZD4, SYNPO2, PLXNA4, DMGDH, HLF, FOS, SLC13A2, KY, NTRK2, TGFBR3, PDE8B, INMT, AK5, EGR3, ANO3, MYRIP, BMPER, SLC4A4, ADAMTS5, EBF2, NAT8L, SCN3B, SAMD5, PGR, HSPA12A, ABCC6, NTN4, SEMA6D, KIAA1377, PDZD2, WISP2, MOCS1, CLSTN2, TSHZ2, CORO2B, TGFBR2, FAM13A, CD34, PARK2, PPP1R12B, SHE, LHFP, GFRA1, CXorf36, NR3C2, PNPLA2, PCDHGB7, CAV2, TBX15, ADRB1, PLCXD3, DTX1, CSRNP1, ANGPTL2, TACC1, DCLK1, RECK, SEMA5A, PODN, TAL1, CRIM1, TPPP, KLF9, PIGR, EGF, RASGRF2, PRICKLE2, PCDHGA2, PID1, AQP1, TMEM88, CNRIP1, CBX7, CD302, UTRN, WSCD1, ATF3, MYO16, PALM2-AKAP2, FMOD, PCSK5, PCDHGB6, CHAD, STC2, FAM189A2, ERG, NMNAT2, SPTBN1, KLF2, MS4A2, OLFML1, SLC29A4, PPAP2A, DACT2, LMO3, TTC28, ANTXR2, LIMS2, C7, TMEM220, PER2, TEF, EZH1, NR4A2, MAPT, KLHL3, C2CD2, KANK3, CDH13, SLC7A2, ZEB2, COL4A3BP, RGS5, VIT, WASF3, PELI2, GREB1, CELF2, KAT2B, PRUNE2, DLK2, HNMT, TWIST2, ARRB1, PKD2, RHOJ, PCDHGA6, PDK2, CDKN1C, EIF4E3, PTGIS, NGFR, RAB30
hsa-miR-21-5p		188		TGFBR3 (MIRT005330), SOX5 (MIRT000960), TP63 (MIRT005329), TGFBR2 (MIRT001189), RECK (MIRT000969), BCL2 (MIRT000159), BTG2 (MIRT002416), FMOD (MIRT001188), SPRY2 (MIRT000672), TIMP3 (MIRT000954)	PER2 (MIRT655270), GPAM (MIRT030803), SAMD5 (MIRT030748), PDGFD (MIRT030746), LIFR (MIRT030857), CYBRD1 (MIRT030691), DDR2 (MIRT030671), APOLD1 (MIRT030891), DMD (MIRT030888), SEMA5A (MIRT030970), KLF9 (MIRT031013), PRICKLE2 (MIRT030840), UTRN (MIRT030742), GPD1L (MIRT030911), FERMT2 (MIRT031065), PPAP2A (MIRT030669), FILIP1L (MIRT031073), TLR4 (MIRT031004), SASH1 (MIRT030781), COBLL1 (MIRT030747), PKD2 (MIRT030759), FOXN3 (MIRT030800)	CSN1S1, ACVR1C, HEPACAM, KLB, HEPN1, CD36, SDPR, OXTR, ABCA10, NOVA1, ABCD2, MAOA, ITIH5, TAT, BMP3, GRIA4, MYH11, HRASLS5, PTGER3, C1QTNF7, SEMA3G, TNS1, COL6A6, GLDN, SLC16A7, LEPR, KL, FZD4, SYNPO2, AKAP12, PLXNA4, DMGDH, CHL1, HMGCS2, NTRK2, PDE8B, ERBB4, LRRC2, EGR3, ANO3, CCDC69, ESR1, PTPRT, RBMS3, ALDH1A1, SCN3B, ABCC9, BHMT2, UGT2B15, CCDC85A, CCDC3, MATN2, TPRG1, SAA2, TMTC1, PTPRB, SCN9A, PDZD2, IL6ST, CLSTN2, FMO2, TSHZ2, PCDH19, RNF157, FGF2, C16orf89, FAM13A, RNASE4, SHE, GFRA1, EMCN, PLN, SCUBE2, CXCL12, FGF1, CLIC5, SOX17, RUNDC3B, LYPD6, THRB, FOXP2, NEGR1, GPR34, GPR116, GPRASP1, ADRBK2, GREM2, RASSF6, EDNRB, HAS3, KLHDC7A, MAST4, DCLK1, AVPR1A, TAL1, AFF3, CRIM1, RETSAT, RASGRF2, NR5A2, ADAMTS18, SPRY1, PID1, AQP1, EDA2R, CNTFR, RNF180, FOXO1, PALM2-AKAP2, CAB39L, GNG2, NR3C1, SPTBN1, ZAK, MS4A2, LCA5, ANO6, LMO3, NECAB1, SPATA18, PARD3B, NXPH3, TTC28, ADAMTS1, MAPK10, ZFPM2, C7, STK32B, PDE1A, SSPN, FAM198B, P2RY14, CCDC152, VGLL3, HSDL2, PLA2R1, CD209, USP53, PHLDB2, PELI2, KCTD12, SOX7, RANBP3L, HNMT, AKR1C2, DLG2, ABCA1, STEAP2, CFL2, DAAM2, PRKG1, PCDH18, PTGIS, C1orf95, PVRL3, RAB30
hsa-miR-181b-5p		337		FOS (MIRT437420), BCL2 (MIRT003500), CBX7 (MIRT006371), TIMP3 (MIRT000637), KAT2B (MIRT004328)	CAT (MIRT047237), FAM13A (MIRT713216), SCD (MIRT055114), PPAP2B (MIRT542774), PER2 (MIRT726983), TEF (MIRT698675)	GLYAT, ATP1A2, KIAA0408, ADIPOQ, TUSC5, GPD1, ADH1B, KCNIP2, SGCG, TRDN, ACVR1C, TMEM132C, SLC19A3, LIPE, HEPN1, GPAM, ACACB, ACSM5, OXTR, SCN4A, GDF10, PDK4, NTS, LPL, TRHDE, CLDN19, AGTR1, PFKFB1, NOVA1, GYG2, GPIHBP1, MAOA, ITIH5, SCN7A, BMP3, MASP1, SMYD1, ARHGAP20, PDE2A, OGN, KCNE1, PDE11A, PDE3B, NAALAD2, HRASLS5, GPX3, PTGER3, C1QTNF7, TNS1, CA3, GLDN, SLC16A7, LUZP2, LEPR, FHL5, SCGB1D2, KLHL31, KL, ATOH8, MAMDC2, SYNPO2, FREM1, DMGDH, HLF, CHL1, KLF15, NTRK2, TGFBR3, LMOD1, ANKRD29, ERBB4, INMT, EBF3, EGR1, EGR3, THRSP, IGFBP6, BMPER, IGF1, CCDC69, SLC4A4, ESR1, ADAMTS5, MME, CXCL2, PKD1L2, PTPRT, EBF2, RBMS3, NAT8L, CNN1, SCN3B, SAMD5, PGR, CYP4F22, BHMT2, HSPA12A, CCDC85A, PDGFD, RAPGEF3, LIFR, THSD7B, MATN2, TPRG1, SAA2, TMTC1, ANGPTL7, PTPRB, NTN4, SOX5, SCN9A, EMX2OS, SEMA6D, GPLD1, SLIT3, PDZD2, RUNX1T1, TP63, IL6ST, DCN, MOCS1, FMO2, IL33, ADRA2A, TSHZ2, AHNAK, STBD1, PCDH19, RNF157, SLC22A3, DST, TGFBR2, DACH1, HSD11B1, PFKFB3, ACSL1, SIK2, PARK2, PLSCR4, HOXA5, ANKRD53, LDB3, PPP1R12B, NAV3, SHE, MEOX1, BCHE, APOLD1, RERGL, GFRA1, EMCN, CXorf36, THSD4, CEBPA, CXCL12, FGF1, SOX17, GSN, THRB, FOXP2, NEGR1, GPR116, S1PR1, CX3CR1, ADRBK2, TFPI, ABI3BP, PLCXD3, GREM2, ARHGEF15, RASSF6, EDNRB, ZCCHC24, MAST4, ANGPTL2, TLN2, DCLK1, RECK, AVPR1A, SEMA5A, ALPK3, ROBO4, AFF3, CRIM1, ITSN1, TPPP, AMIGO2, KLF9, DIXDC1, MSRB3, RASGRF2, NR5A2, PRICKLE2, ADAMTS18, ELTD1, SEMA3D, SEMA3C, AASS, BTG2, PDE5A, FRY, DLC1, CD302, EGR2, SYNM, PKDCC, WSCD1, GNAL, SOCS2, ATF3, GNAI1, PALM2-AKAP2, SLIT2, PCSK5, PDE1B, GNG2, NR3C1, STC2, NR4A3, ARHGEF6, ERG, ZNF423, NMNAT2, KLHL29, TMEM26, SPTBN1, ZAK, KCNB1, RSPO3, LRP1, MS4A2, STX11, CACHD1, GPD1L, ANO6, TTC18, TACR1, DNALI1, EPDR1, CPM, LMO3, NECAB1, SPATA18, TBC1D9, PARD3B, PPP1R3C, DYNC2H1, CRY2, SYN2, TTC28, ARHGAP6, ANTXR2, ADAMTS1, SYNE1, MAPK10, MYLK, CDH23, STS, DSEL, TRPC6, PDE1A, CRYBG3, TMEM170B, NOTCH4, ST6GALNAC3, RYR3, EZH1, TLL1, LOC401052, VGLL3, MAPT, TTBK2, HSDL2, FAM89A, KLHL3, C2CD2, PRTG, CDH13, ZEB1, RGS6, SLC7A2, ZEB2, IRS1, ABCA5, PHLDB2, ACSS3, F3, RGS5, TMEM47, WASF3, INPP4B, PELI2, GREB1, TLR4, SASH1, KCTD12, SOX7, MITF, CYP2U1, PRUNE2, DLK2, CADM3, CDC14B, DLG2, ARID5B, ABCA1, ARRB1, STEAP2, ANGPT1, PKD2, PRKD1, LRRK2, CFL2, RNF125, RHOJ, FYCO1, PRKG1, MMP28, PDK2, C10orf10, RTN1, CDON, PTGIS, PVRL3, EGFLAM, RAB30
hsa-miR-183-5p		257		FOXO1 (MIRT003298), AKAP12 (MIRT005870), EGR1 (MIRT006540), ZEB1 (MIRT054653), DKK3 (MIRT053048)	SH3D19 (MIRT047081), NHSL2 (MIRT047043), KY (MIRT526274), HOXA5 (MIRT025008), CRIM1 (MIRT538391), NR3C1 (MIRT378074), TMEM170B (MIRT565335), PDP2 (MIRT632602), C16orf45 (MIRT664359), TXNIP (MIRT025014)	DEFB132, ATP1A2, PLIN1, TIMP4, CD300LG, AQPEP, C14orf180, CSN1S1, SGCG, TRDN, ACVR1C, HEPACAM, SLC2A4, KLB, NPY2R, SLC19A3, GPAM, ABCA8, LYVE1, RDH5, ALDH1L1, NNAT, PDK4, TRHDE, CLDN19, PFKFB1, NOVA1, ABCD2, MAOA, SORBS1, GLRA3, MASP1, KCNE1, PDE11A, PDE3B, PPP1R1A, HRASLS5, PRRT4, PTGER3, C1QTNF7, TNS1, PCOLCE2, SLC16A7, ADCY5, ALDH1A2, CAV1, KL, SCN3A, ATOH8, HIF3A, FZD4, SYNPO2, FREM1, PLXNA4, DMGDH, XPNPEP2, HLF, HMGCS2, CAPN11, PGM5, NTRK2, PLIN5, LMOD1, PDE8B, EBF3, AK5, MYRIP, IGF1, MEOX2, SLC4A4, ADAMTS5, MME, GIPC2, PTPRT, COL17A1, SCN3B, ABCC9, PGR, PDGFD, LIFR, THSD7B, TMTC1, CMYA5, NTN4, SOX5, SCN9A, KIT, EMX2OS, SEMA6D, SLIT3, KIAA1377, PDZD2, CYBRD1, RUNX1T1, ITPR1, IL6ST, DCN, MOCS1, FMO2, LRRC70, GFAP, CORO2B, STBD1, RAI2, PCDH19, RNF157, DST, PLSCR4, PPP1R12B, NAV3, PEAR1, PHYHIP, APOLD1, RERGL, GFRA1, THSD4, CXCL12, CLIC5, SH3BGRL2, ADAMTS15, DMRT2, GPR34, ACER2, SCD, ADRBK2, LRP1B, EPAS1, PLCXD3, GREM2, ACO1, CSRNP1, ZCCHC24, TACC1, DCLK1, RECK, SEMA5A, ALPK3, ROBO4, TAL1, F8, AMIGO2, DIXDC1, RASGRF2, PRICKLE2, KLHL13, EDA2R, SEMA3D, C4orf32, PDE5A, CNRIP1, DLC1, SYNM, GNAL, SOCS2, GRPR, FMOD, CDH5, PCSK5, PLEKHH2, CAB39L, NR4A3, NMNAT2, KLHL29, TMEM26, SPTBN1, ZAK, EFEMP1, CACHD1, LCA5, ANO6, TACR1, DNALI1, EPDR1, IL20RA, CPM, LMO3, SPATA18, PARD3B, PPP1R3C, SYN2, EFCAB1, NXPH3, ARHGAP6, ANTXR2, LIMS2, FAT4, ZFPM2, C3orf55, TRPC6, PDE1A, FAM198B, ST6GALNAC3, TEF, EZH1, NR4A2, VGLL3, MAPT, MCAM, HSDL2, FAM89A, SPRY2, KLHL3, PRTG, CD209, RGS6, SLC7A2, TIMP3, ZEB2, IRS1, PHLDB2, RGS5, ABCC8, CELF2, KCTD12, PECR, MITF, KAT2B, PRUNE2, COBLL1, CDC14B, TWIST2, DLG2, ABCA1, AIFM2, ARRB1, STEAP2, ANGPT1, PKD2, PRKD1, CFL2, FOXN3, RNF125, FYCO1, PRKG1, PDK2, LRIG1, CDKN1C, CDON, C1orf95, NGFR, PVRL3, RAB30

Supplementary Table S4. Characteristics of the selected target genes.

Target gene	Sample Size		Normal vs Tumor			Curated miRNA(s)
	Normal	Tumor	BaseMean	log2FC	WPS *p* -value	
TNS1	87	117	33454.10684	-3.485642769	3.58E-126	hsa-miR-210-3p, hsa-miR-21-5p, hsa-miR-181b-5p, hsa-miR-183-5p
DCLK1	87	117	1655.874159	-2.11642326	2.82E-31	hsa-miR-210-3p, hsa-miR-21-5p, hsa-miR-181b-5p, hsa-miR-183-5p
SEMA5A	87	117	3359.863653	-2.090182218	1.74E-33	hsa-miR-210-3p, hsa-miR-21-5p, hsa-miR-181b-5p, hsa-miR-183-5p
ACVR1C	87	117	2192.919527	-5.404092925	4.30E-113	hsa-miR-210-3p, hsa-miR-21-5p, hsa-miR-181b-5p, hsa-miR-183-5p
NTRK2	87	117	10867.35029	-3.026831859	2.72E-45	hsa-miR-210-3p, hsa-miR-21-5p, hsa-miR-181b-5p, hsa-miR-183-5p
TGFBR2	87	117	14639.95147	-2.449050735	1.26E-86	hsa-miR-210-3p, hsa-miR-21-5p, hsa-miR-181b-5p
ALDH1A1	87	117	3984.034053	-2.761963644	1.40E-56	hsa-miR-21-5p
NMNAT2	87	117	551.0337988	-1.916125391	3.76E-19	hsa-miR-210-3p, hsa-miR-181b-5p, hsa-miR-183-5p

Supplementary Table S5. Official full name of the selected target genes.

Gene Symbol	Official Full Name
TNS1	tensin 1
DCLK1	doublecortin like kinase 1
SEMA5A	semaphorin 5A
ACVR1C	activin A receptor type 1C
NTRK2	neurotrophic receptor tyrosine kinase 2
TGFBR2	transforming growth factor beta receptor II
ALDH1A1	aldehyde dehydrogenase 1 family member A1
NMNAT2	nicotinamide nucleotide adenylyltransferase 2

Supplementary Table S6. Information of computationally predicted hsa-miR-210-3p binding sites in 3'-UTRs of target.

<table>
<tr><th>MiRNA</th><th>Target Gene</th><th>Target Site</th><th>Start-End</th><th>Seed Type</th><th>Score (MiRanda)</th><th>MFE (MiRanda)</th><th>Prediction Tool</th></tr>
<tr><td rowspan="9">miR-210</td><td>TNS1-2 √</td><td>miRNA: 3' agucgGCGACAGUG--UGCGUGUc 5'
               :|| ||| |||||||
Target:5' acccaTGCACACACCTACGCACAc 3'</td><td>2738-2765</td><td>7mer-m8</td><td>144</td><td>-15.54</td><td>TS, PT, MR</td></tr>
<tr><td>DCLK1-1 √</td><td>miRNA: 3' agUCGGCGACAGUGUGCGUGUc 5'
            | |: | | |||||||||:
Target:5' atAACTTC-GACACACGCACGg 3'</td><td>612-645</td><td>none</td><td>142</td><td>-24.879999</td><td>RH, PT, MR</td></tr>
<tr><td>DCLK1-1</td><td>miRNA: 3' agUCGGCGACAGUGUGCGUGuc 5'
            | |:||| |: |||||||
Target:5' tcACCTGCTTTTCCACGCACtg 3'</td><td>1043-1065</td><td>none</td><td>148</td><td>-23.52</td><td>RH, PT, MR</td></tr>
<tr><td>SEMA5A-1</td><td>miRNA: 3' agUC-GGCGACAG-UGUGCGUGUc 5'
            || |: ||||| |:|:|||||
Target:5' agAGTCTTCTGTCTATATGCACAt 3'</td><td>1866-1895</td><td>none</td><td>150</td><td>-23.4</td><td>RH, PT, MR</td></tr>
<tr><td>SEMA5A-2 √</td><td>miRNA: 3' agucggcgacaguguGCGUGUc 5'
                         ||||||
Target:5' gtggtcaggctccacCGCACAg 3'</td><td>3156-3184</td><td>6mer</td><td>120</td><td>-23.75</td><td>RH, PT, MR</td></tr>
<tr><td>ACVR1C-1 √</td><td>miRNA: 3' agucggcgacaguguGCGUGUc 5'
                         ||||||
Target:5' gaatcataatggaccCGCACAa 3'</td><td>859-881</td><td>7mer-A1</td><td>120</td><td>-12.12</td><td>TS, PT, MR</td></tr>
<tr><td>TGFBR2-1 √</td><td>miRNA: 3' agucGGCGACAGUGUGCGUGUc 5'
              |:| | | || |||||
Target:5' gctcCTGATTGCTCAAGCACAg 3'</td><td>1062-1086</td><td>none</td><td>122</td><td>-19.09</td><td>RH, PT, MR</td></tr>
<tr><td>NMNAT2-1 √</td><td>miRNA: 3' agUCGGCGACAGUGUGCGUGUc 5'
            ||| | ||||||||||
Target:5' tcAGCACCACACACACGCACAc 3'</td><td>676-716</td><td>7mer-m8</td><td>160</td><td>-24.84</td><td>RH, TS, PT, MR</td></tr>
<tr><td>NMNAT2-1</td><td>miRNA: 3' aguCGGCGACAGUGUGCGUGUc 5'
             || | | |:||||||||
Target:5' tgtGCACCCG-CGCACGCACAt 3'</td><td>718-754</td><td>7mer-m8</td><td>153</td><td>-23.06</td><td>RH, TS, PT, MR</td></tr>
</table>

* Selected mRNA target sequences were marked with red marker.

Supplementary Table S7. Information of computationally predicted hsa-miR-21-5p binding sites in 3'-UTRs of target genes.

<table>
<tr><th>MiRNA</th><th>Target Gene</th><th>Target Site</th><th>Start-End</th><th>Seed Type</th><th>Score (MiRanda)</th><th>MFE (MiRanda)</th><th>Prediction Tool</th></tr>
<tr><td rowspan="14">miR-21</td><td>TNS1-1 √</td><td>miRNA: 3' agUUGUAGUCAGACUAUUCGAu 5'
            :||| | ||| :||||||
Target:5' ctGACA-GAATCT-GTAAGCTa 3'</td><td>1629-1651</td><td>7mer-A1</td><td>136</td><td>-16.280001</td><td>TS, PT, MR</td></tr>
<tr><td>DCLK-2 √</td><td>miRNA: 3' aguuguagucagacuAUUCGAu 5'
                         ||||||
Target:5' caatctttctaacatTAAGCTa 3'</td><td>2576-2598</td><td>7mer-A1</td><td>120</td><td>-10.65</td><td>TS, PT, MR</td></tr>
<tr><td>SEMA5A-1</td><td>miRNA: 3' aguugUAGUCAGACUAUUCGau 5'
               |||| || |||||||
Target:5' cactgATCA-TC-GATAAGCat 3'</td><td>1348-1367</td><td>none</td><td>137</td><td>-12.64</td><td>PT, MR</td></tr>
<tr><td>SEMA5A-1</td><td>miRNA: 3' aguuGUAGUCAGACUAUUCGAu 5'
              :| :|||:| :||||||
Target:5' tcccTAATAGTTT-GTAAGCTt 3'</td><td>2051-2071</td><td>6mer</td><td>140</td><td>-15.97</td><td>PT, MR</td></tr>
<tr><td>SEMA5A-1</td><td>miRNA: 3' agUUGUAGUCAGACUAUUCGAu 5'
            | || | || |||||:|
Target:5' ttACCAGCTGTGGAATAAGTTg 3'</td><td>2403-2424</td><td>none</td><td>136</td><td>-11.71</td><td>PT, MR</td></tr>
<tr><td>SEMA5A-2</td><td>miRNA: 3' agUUGUAGUCAG--ACUAUUCGAu 5'
            ||:| ||||: ||||:|||
Target:5' tgAATA-CAGTTGGAGATAGGCTg 3'</td><td>2856-2879</td><td>none</td><td>141</td><td>-18.549999</td><td>RH, PT, MR</td></tr>
<tr><td>SEMA5A-3 √</td><td>miRNA: 3' agUUGUAGUCAG--ACUAUUCGAu 5'
            ||: |||| | ||:||||||
Target:5' ccAATTTCAGCCCTTGGTAAGCTt 3'</td><td>6955-6978</td><td>6mer</td><td>151</td><td>-20.74</td><td>RH, PT, MR</td></tr>
<tr><td>ACVR1C-1 √</td><td>miRNA: 3' aguUGUAGUCAGA--CUAUUCGAu 5'
             ||| ||| | |||||||
Target:5' attACAAAAGTATAAAATAAGCTc 3'</td><td>263-286</td><td>7mer-m8</td><td>150</td><td>-11.07</td><td>TS, PT, MR</td></tr>
<tr><td>ACVR1C-3</td><td>miRNA: 3' aguuguagucAGACUAUUCGAu 5'
                    |:| :||||||
Target:5' tgtaaagactTTTTGTAAGCTa 3'</td><td>6547-6569</td><td>7mer-A1</td><td>132</td><td>-10.88</td><td>TS, PT, MR</td></tr>
<tr><td>NTRK2-1 √</td><td>miRNA: 3' aguuguagucagacUAUUCGAu 5'
                        |||||||
Target:5' cctgcactttaaaaATAAGCTa 3'</td><td>1488-1510</td><td>8mer</td><td>140</td><td>-8.81</td><td>TS, PT, MR</td></tr>
<tr><td>TGFBR2-1 √</td><td>miRNA: 3' agUUGUAGUCAG-A--CUAUUCGAu 5'
            :||||: ||| | ||||||||
Target:5' ttGACATT-GTCATAGGATAAGCTg 3'</td><td>189-214</td><td>7mer-m8</td><td>156</td><td>-19.610001</td><td>RH, TS, PT, MR</td></tr>
<tr><td>TGFBR2-1</td><td>miRNA: 3' agUUGUAGUCAGACUAUUCGAu 5'
            || | || | ||:||||
Target:5' acAAGAACAAAGT-ATGAGCTt 3'</td><td>1746-1766</td><td>none</td><td>130</td><td>-10.65</td><td>PT, MR</td></tr>
<tr><td>ALDH1A1-1 √</td><td>miRNA: 3' aguuguagucagacUAUUCGAu 5'
                        |||||||
Target:5' tgatttttttaaacATAAGCTa 3'</td><td>95-117</td><td>8mer</td><td>140</td><td>-7.97</td><td>TS, PT, MR</td></tr>
</table>

* Selected mRNA target sequences were marked with red marker.

Supplementary Table S8. Information of computationally predicted hsa-miR-181b-5p binding sites in 3’-UTRs of target genes.

<table>
<tr><th>MiRNA</th><th>Target Gene</th><th>Target Site</th><th>Start-End</th><th>Seed Type</th><th>Score (MiRanda)</th><th>MFE (MiRanda)</th><th>Prediction Tool</th></tr>
<tr><td rowspan="9">miR-181b</td><td>TNS1-2 √</td><td>miRNA: 3' ugGGUGGCUGUCGUUACUUACAa 5'
::|:| ::| ||||||||
Target:5' ccTTATC-CTGGACATGAATGTa 3'</td><td>4553-4575</td><td>8mer</td><td>147</td><td>-14.43</td><td>TS, PT, MR</td></tr>
<tr><td>DCLK1-1 √</td><td>miRNA: 3' ugggUGGCU-GUCGUUACUUACAa 5'
||: | ||||||||||||
Target:5' ttaaACTTAGAAGCAATGAATGTt 3'</td><td>358-381</td><td>7mer-m8</td><td>166</td><td>-20.639999</td><td>TS, PT, MR</td></tr>
<tr><td>DCLK1-2</td><td>miRNA: 3' ugGGUGGCUGUCGUUACUUACAa 5'
|:||| | | | |||||||
Target:5' agCTACCAAGATC-CTGAATGTc 3'</td><td>2592-2614</td><td>7mer-m8</td><td>155</td><td>-15.85</td><td>TS, PT, MR</td></tr>
<tr><td>DCLK1-2</td><td>miRNA: 3' ugggugcUGUCGUUACUUACaa 5'
|:| | |||||||
Target:5' aaagcaatATATCCATGAATGaa 3'</td><td>3365-3410</td><td>none</td><td>135</td><td>-8.83</td><td>RH, PT, MR</td></tr>
<tr><td>SEMA5A-3 √</td><td>miRNA: 3' ugGGUGGCUGUCGU---UACUUACAa 5'
|:|:| ||||| |||:|||
Target:5' atCTATCCTCAGCACTGCTGAGTGTg 3'</td><td>7650-7676</td><td>none</td><td>140</td><td>-21.32</td><td>RH, PT, MR</td></tr>
<tr><td>ACVR1C-3 √</td><td>miRNA: 3' ugGGUGGCUGUC---G-UUACUUACAa 5'
| :||||||| | || ||||||
Target:5' ttCAGCCGACAGGCCCAAAGGAATGTa 3'</td><td>6602-6629</td><td>7mer-A1</td><td>135</td><td>-22.83</td><td>RH, TS, PT, MR</td></tr>
<tr><td>TGFBR2-1 √</td><td>miRNA: 3' ugGGUGGCUGUCGU--UACUUACAa 5'
|||:| :| :| |||||||
Target:5' ccCCATCTTTAATACCTTGAATGTt 3'</td><td>771-795</td><td>7mer-m8</td><td>148</td><td>-19.02</td><td>TS, PT, MR</td></tr>
<tr><td>TGFBR2-1</td><td>miRNA: 3' ugGGUGG-CUGUCGU-UACUUACAa 5'
::||: || || | |||||||
Target:5' aaTTACTAGAGAGGATTTGAATGTg 3'</td><td>1675-1699</td><td>7mer-m8</td><td>151</td><td>-15.7</td><td>TS, PT, MR</td></tr>
<tr><td>TGFBR2-1</td><td>miRNA: 3' ugggugcugucguuaCUUACAa 5'
||||||
Target:5' ttatcagcataaactgGAATGTa 3'</td><td>2183-2206</td><td>7mer-A1</td><td>120</td><td>-11.69</td><td>TS, PT, MR</td></tr>
</table>

* Selected mRNA target sequences were marked with red marker.

Supplementary Table S9. Information of computationally predicted hsa-miR-183-5p binding sites in 3’-UTRs of target genes.

<table>
<tr><th>MiRNA</th><th>Target Gene</th><th>Target Site</th><th>Start-End</th><th>Seed Type</th><th>Score (MiRanda)</th><th>MFE (MiRanda)</th><th>Prediction Tool</th></tr>
<tr><td rowspan="6">miR-183</td><td>DCLK-1 √</td><td>miRNA: 3' ucacuuaAGAUG-GUCACGGUau 5'
||||| |||||:||
Target:5' gggtctcTCTACTCAGTGTCAga 3'</td><td>905-937</td><td>none</td><td>130</td><td>-19.370001</td><td>RH, PT, MR</td></tr>
<tr><td>SEMA5A-1</td><td>miRNA: 3' ucACUUAAGAUGGUCACGGUAu 5'
|| | |:| | |||||||
Target:5' gaTGCAGTTT--CTGTGCCATt 3'</td><td>627-649</td><td>7mer-m8</td><td>149</td><td>-13.62</td><td>TS, PT, MR</td></tr>
<tr><td>SEMA5A-2 √</td><td>miRNA: 3' ucacuuaaGAUGGUCACGGUAu 5'
|| |||||||:||
Target:5' gaaagtggCTCCCAGTGCTATt 3'</td><td>2928-2949</td><td>none</td><td>146</td><td>-23.280001</td><td>RH, PT, MR</td></tr>
<tr><td>SEMA5A-2</td><td>miRNA: 3' ucacuuaaGAUGG---UCACGGUau 5'
||:|| |||||||
Target:5' aggatcaaCTGCCCAGAGTGCCAga 3'</td><td>3704-3728</td><td>none</td><td>129</td><td>-18.959999</td><td>RH, PT, MR</td></tr>
<tr><td>SEMA5A-3</td><td>miRNA: 3' ucacuuaagauggucACGGUAu 5'
||||||
Target:5' atattagctcatatcTGCCATa 3'</td><td>5581-5603</td><td>7mer-A1</td><td>120</td><td>-12.2</td><td>TS, PT, MR</td></tr>
<tr><td>NTRK2-1 √</td><td>miRNA: 3' ucacUUAAGA-UGGU----CACGGUAu 5'
||||:| ||| |||||||
Target:5' taaaAATTTTCTCCAGAGTGTGCCATa 3'</td><td>831-857</td><td>8mer</td><td>148</td><td>-17.530001</td><td>TS, PT, MR</td></tr>
</table>

* Selected mRNA target sequences were marked with red marker.

Supplementary Table S10. Identified double inhibitory feed-forward loops from TF-miRNA-mRNA network in TNBC.

TF	miRNA	miRNA	mRNA
JUN	hsa-miR-183-5p	hsa-miR-21-5p	NTRK2
JUND	hsa-miR-183-5p	hsa-miR-21-5p	NTRK2
TFAP4	hsa-miR-183-5p	hsa-miR-21-5p	NTRK2
TFAP2C	hsa-miR-183-5p	hsa-miR-21-5p	NTRK2
SOX2	hsa-miR-183-5p	hsa-miR-21-5p	NTRK2
NR2F2	hsa-miR-183-5p	hsa-miR-21-5p	NTRK2
NANOG	hsa-miR-183-5p	hsa-miR-21-5p	NTRK2
KLF4	hsa-miR-183-5p	hsa-miR-21-5p	NTRK2
HNF4A	hsa-miR-183-5p	hsa-miR-21-5p	NTRK2
HDAC2	hsa-miR-183-5p	hsa-miR-21-5p	NTRK2
GTF2I	hsa-miR-183-5p	hsa-miR-21-5p	NTRK2
CEBPB	hsa-miR-183-5p	hsa-miR-21-5p	NTRK2
BRPF3	hsa-miR-183-5p	hsa-miR-21-5p	NTRK2
ASXL1	hsa-miR-183-5p	hsa-miR-21-5p	NTRK2
ZBTB7A	hsa-miR-183-5p	hsa-miR-21-5p	NTRK2
TRIM28	hsa-miR-183-5p	hsa-miR-21-5p	NTRK2
TFAP2A	hsa-miR-183-5p	hsa-miR-21-5p	NTRK2
TCF3	hsa-miR-183-5p	hsa-miR-21-5p	NTRK2
STAT3	hsa-miR-183-5p	hsa-miR-21-5p	NTRK2
RELA	hsa-miR-183-5p	hsa-miR-21-5p	NTRK2
NIPBL	hsa-miR-183-5p	hsa-miR-21-5p	NTRK2
MYCN	hsa-miR-183-5p	hsa-miR-21-5p	NTRK2
MYC	hsa-miR-183-5p	hsa-miR-21-5p	NTRK2
MED1	hsa-miR-183-5p	hsa-miR-21-5p	NTRK2
MBD3	hsa-miR-183-5p	hsa-miR-21-5p	NTRK2
MAX	hsa-miR-183-5p	hsa-miR-21-5p	NTRK2
KLF5	hsa-miR-183-5p	hsa-miR-21-5p	NTRK2
HIF1A	hsa-miR-183-5p	hsa-miR-21-5p	NTRK2
ESR1	hsa-miR-183-5p	hsa-miR-21-5p	NTRK2
ERG	hsa-miR-183-5p	hsa-miR-21-5p	NTRK2
EP300	hsa-miR-183-5p	hsa-miR-21-5p	NTRK2
EGR1	hsa-miR-183-5p	hsa-miR-21-5p	NTRK2
E2F1	hsa-miR-183-5p	hsa-miR-21-5p	NTRK2
CTCF	hsa-miR-183-5p	hsa-miR-21-5p	NTRK2
CREB1	hsa-miR-183-5p	hsa-miR-21-5p	NTRK2
BRD4	hsa-miR-183-5p	hsa-miR-21-5p	NTRK2
AR	hsa-miR-183-5p	hsa-miR-21-5p	NTRK2
SNAI2	hsa-miR-183-5p	hsa-miR-210-3p	SEMA5A
MYOD11	hsa-miR-183-5p	hsa-miR-210-3p	SEMA5A
JUN	hsa-miR-183-5p	hsa-miR-210-3p	SEMA5A

JUND	hsa-miR-183-5p	hsa-miR-210-3p	SEMA5A
ZBTB7A	hsa-miR-183-5p	hsa-miR-210-3p	SEMA5A
TRIM28	hsa-miR-183-5p	hsa-miR-210-3p	SEMA5A
TRIM24	hsa-miR-183-5p	hsa-miR-210-3p	SEMA5A
TFAP2A	hsa-miR-183-5p	hsa-miR-210-3p	SEMA5A
TCF3	hsa-miR-183-5p	hsa-miR-210-3p	SEMA5A
STAT3	hsa-miR-183-5p	hsa-miR-210-3p	SEMA5A
SP2	hsa-miR-183-5p	hsa-miR-210-3p	SEMA5A
RELA	hsa-miR-183-5p	hsa-miR-210-3p	SEMA5A
NRF1	hsa-miR-183-5p	hsa-miR-210-3p	SEMA5A
NR2F1	hsa-miR-183-5p	hsa-miR-210-3p	SEMA5A
NIPBL	hsa-miR-183-5p	hsa-miR-210-3p	SEMA5A
NFYB	hsa-miR-183-5p	hsa-miR-210-3p	SEMA5A
NFYA	hsa-miR-183-5p	hsa-miR-210-3p	SEMA5A
MYCN	hsa-miR-183-5p	hsa-miR-210-3p	SEMA5A
MYC	hsa-miR-183-5p	hsa-miR-210-3p	SEMA5A
MED1	hsa-miR-183-5p	hsa-miR-210-3p	SEMA5A
MBD3	hsa-miR-183-5p	hsa-miR-210-3p	SEMA5A
MAX	hsa-miR-183-5p	hsa-miR-210-3p	SEMA5A
KLF5	hsa-miR-183-5p	hsa-miR-210-3p	SEMA5A
HIF1A	hsa-miR-183-5p	hsa-miR-210-3p	SEMA5A
ESR1	hsa-miR-183-5p	hsa-miR-210-3p	SEMA5A
ERG	hsa-miR-183-5p	hsa-miR-210-3p	SEMA5A
EP300	hsa-miR-183-5p	hsa-miR-210-3p	SEMA5A
EGR1	hsa-miR-183-5p	hsa-miR-210-3p	SEMA5A
E2F1	hsa-miR-183-5p	hsa-miR-210-3p	SEMA5A
CTCF	hsa-miR-183-5p	hsa-miR-210-3p	SEMA5A
CREB1	hsa-miR-183-5p	hsa-miR-210-3p	SEMA5A
BRD4	hsa-miR-183-5p	hsa-miR-210-3p	SEMA5A
AR	hsa-miR-183-5p	hsa-miR-210-3p	SEMA5A
STAT3	hsa-miR-210-3p	hsa-miR-181b-5p	SEMA5A

Supplementary Table S11. Total of 66 significant KEGG pathways (p-value <0.05).

ID	Description	GeneRatio	BgRatio	pvalue	p.adjust	qvalue	geneID	Count
hsa05207	Chemical carcinogenesis - receptor activation	10/31	212/8223	2.90198E-09	4.14983E-07	2.35213E-07	JUN/STAT3/RELA/MYC/KLF5/ESR1/E2F1/CREB1/AR/KLF4	10
hsa05166	Human T-cell leukemia virus 1 infection	9/31	222/8223	7.79216E-08	5.5714E-06	3.15788E-06	JUN/TCF3/RELA/NFYB/MYC/EP300/EGR1/E2F1/CREB1	9
hsa05202	Transcriptional misregulation in cancer	8/31	193/8223	3.95573E-07	1.47128E-05	8.33922E-06	TCF3/RELA/MYCN/MYC/MAX/ERG/HDAC2/CEBPB	8
hsa05167	Kaposi sarcoma-associated herpesvirus infection	8/31	194/8223	4.11546E-07	1.47128E-05	8.33922E-06	JUN/STAT3/RELA/MYC/HIF1A/EP300/E2F1/CREB1	8
hsa05215	Prostate cancer	6/31	97/8223	1.33778E-06	3.82605E-05	2.16861E-05	RELA/ERG/EP300/E2F1/CREB1/AR	6
hsa05161	Hepatitis B	7/31	162/8223	1.78715E-06	4.25937E-05	2.41422E-05	JUN/STAT3/RELA/MYC/EP300/E2F1/CREB1	7
hsa04919	Thyroid hormone signaling pathway	6/31	121/8223	4.88541E-06	9.9802E-05	5.65679E-05	MYC/MED1/HIF1A/ESR1/EP300/HDAC2	6
hsa04550	Signaling pathways regulating pluripotency of stem cells	6/31	143/8223	1.28122E-05	0.000229017	0.000129807	TCF3/STAT3/MYC/SOX2/NANOG/KLF4	6
hsa05152	Tuberculosis	6/31	180/8223	4.72658E-05	0.000751001	0.000425669	RELA/NFYB/NFYA/EP300/CREB1/CEBPB	6
hsa05169	Epstein-Barr virus infection	6/31	202/8223	8.99221E-05	0.001162739	0.000659043	JUN/STAT3/RELA/MYC/E2F1/HDAC2	6
hsa05203	Viral carcinogenesis	6/31	204/8223	9.49668E-05	0.001162739	0.000659043	JUN/STAT3/RELA/EP300/CREB1/HDAC2	6
hsa05205	Proteoglycans in cancer	6/31	205/8223	9.75725E-05	0.001162739	0.000659043	STAT3/MYC/HIF1A/ESR1/TFAP4/NANOG	6
hsa04137	Mitophagy - animal	4/31	72/8223	0.000142192	0.001564112	0.000886541	JUN/RELA/HIF1A/E2F1	4
hsa05220	Chronic myeloid leukemia	4/31	76/8223	0.000175468	0.001792285	0.00101587	RELA/MYC/E2F1/HDAC2	4
hsa05235	PD-L1 expression and PD-1 checkpoint pathway in cancer	4/31	89/8223	0.000322714	0.003076536	0.001743786	JUN/STAT3/RELA/HIF1A	4
hsa05222	Small cell lung cancer	4/31	92/8223	0.000366407	0.003274763	0.001856141	RELA/MYC/MAX/E2F1	4
hsa04657	IL-17 signaling pathway	4/31	94/8223	0.000397795	0.003346155	0.001896606	JUN/JUND/RELA/CEBPB	4
hsa01522	Endocrine resistance	4/31	98/8223	0.000466279	0.003704326	0.002099618	JUN/MED1/ESR1/E2F1	4
hsa04933	AGE-RAGE signaling pathway in diabetic complications	4/31	100/8223	0.000503504	0.003789532	0.002147913	JUN/STAT3/RELA/EGR1	4
hsa04659	Th17 cell differentiation	4/31	108/8223	0.000673818	0.004750672	0.002692688	JUN/STAT3/RELA/HIF1A	4
hsa04066	HIF-1 signaling pathway	4/31	109/8223	0.000697651	0.004750672	0.002692688	STAT3/RELA/HIF1A/EP300	4
hsa05030	Cocaine addiction	3/31	49/8223	0.000794908	0.005134818	0.002910423	JUN/RELA/CREB1	3
hsa04668	TNF signaling pathway	4/31	114/8223	0.00082588	0.005134818	0.002910423	JUN/RELA/CREB1/CEBPB	4
hsa04110	Cell cycle	4/31	127/8223	0.001236136	0.007279637	0.00412611	MYC/EP300/E2F1/HDAC2	4
hsa04380	Osteoclast differentiation	4/31	128/8223	0.001272664	0.007279637	0.00412611	JUN/JUND/RELA/CREB1	4
hsa05163	Human cytomegalovirus infection	5/31	225/8223	0.001393721	0.007665466	0.004344798	STAT3/RELA/MYC/E2F1/CREB1	5
hsa05321	Inflammatory bowel disease	3/31	65/8223	0.001809318	0.009582683	0.00543148	JUN/STAT3/RELA	3
hsa05221	Acute myeloid leukemia	3/31	67/8223	0.00197425	0.009909839	0.005616913	STAT3/RELA/MYC	3
hsa05224	Breast cancer	4/31	147/8223	0.002119093	0.009909839	0.005616913	JUN/MYC/ESR1/E2F1	4
hsa05031	Amphetamine addiction	3/31	69/8223	0.002148287	0.009909839	0.005616913	JUN/CREB1/HDAC2	3
hsa05211	Renal cell carcinoma	3/31	69/8223	0.002148287	0.009909839	0.005616913	JUN/HIF1A/EP300	3
hsa04917	Prolactin signaling pathway	3/31	70/8223	0.002238775	0.010004524	0.00567058	STAT3/RELA/ESR1	3
hsa05160	Hepatitis C	4/31	157/8223	0.002692926	0.011669346	0.006614204	STAT3/RELA/MYC/E2F1	4
hsa05212	Pancreatic cancer	3/31	76/8223	0.002831601	0.011909379	0.006750255	STAT3/RELA/E2F1	3
hsa04612	Antigen processing and presentation	3/31	78/8223	0.003048705	0.012456139	0.00706016	NFYB/NFYA/CREB1	3
hsa04010	MAPK signaling pathway	5/31	294/8223	0.00446749	0.017745863	0.010058384	JUN/JUND/RELA/MYC/MAX	5
hsa05206	MicroRNAs in cancer	5/31	310/8223	0.005590597	0.021606903	0.012246828	STAT3/MYC/EP300/E2F1/HDAC2	5
hsa04928	Parathyroid hormone synthesis, secretion and action	3/31	106/8223	0.007200481	0.026961447	0.015281792	JUND/EGR1/CREB1	3
hsa05165	Human papillomavirus infection	5/31	331/8223	0.007353122	0.026961447	0.015281792	RELA/EP300/E2F1/CREB1/HDAC2	5
hsa04931	Insulin resistance	3/31	108/8223	0.007581295	0.02710313	0.015362098	STAT3/RELA/CREB1	3
hsa04024	cAMP signaling pathway	4/31	225/8223	0.009615837	0.033538163	0.019009485	JUN/RELA/EP300/CREB1	4
hsa04935	Growth hormone synthesis, secretion and action	3/31	120/8223	0.010116511	0.034232179	0.019402854	STAT3/EP300/CREB1	3
hsa05219	Bladder cancer	2/31	41/8223	0.010293592	0.034232179	0.019402854	MYC/E2F1	2
hsa04926	Relaxin signaling pathway	3/31	129/8223	0.012306275	0.039995392	0.022669453	JUN/RELA/CREB1	3
hsa04915	Estrogen signaling pathway	3/31	138/8223	0.014749352	0.046744314	0.026494753	JUN/ESR1/CREB1	3
hsa05162	Measles	3/31	139/8223	0.015036633	0.046744314	0.026494753	JUN/STAT3/RELA	3
hsa04218	Cellular senescence	3/31	156/8223	0.020410789	0.060573465	0.034333138	RELA/MYC/E2F1	3
hsa04330	Notch signaling pathway	2/31	59/8223	0.020595901	0.060573465	0.034333138	EP300/HDAC2	2
hsa04390	Hippo signaling pathway	3/31	157/8223	0.020755943	0.060573465	0.034333138	SNAI2/MYC/SOX2	3
hsa04630	JAK-STAT signaling pathway	3/31	166/8223	0.024008274	0.068663663	0.038918675	STAT3/MYC/EP300	3
hsa04310	Wnt signaling pathway	3/31	170/8223	0.025538183	0.071607063	0.040586999	JUN/MYC/EP300	3
hsa05016	Huntington disease	4/31	306/8223	0.026804588	0.073675117	0.041759176	NRF1/EP300/CREB1/HDAC2	4
hsa04920	Adipocytokine signaling pathway	2/31	69/8223	0.027590932	0.073675117	0.041759176	STAT3/RELA	2
hsa05120	Epithelial cell signaling in Helicobacter pylori infection	2/31	70/8223	0.028336584	0.073675117	0.041759176	JUN/RELA	2
hsa05230	Central carbon metabolism in cancer	2/31	70/8223	0.028336584	0.073675117	0.041759176	MYC/HIF1A	2
hsa04520	Adherens junction	2/31	71/8223	0.029090351	0.074284289	0.042104455	SNAI2/EP300	2
hsa05223	Non-small cell lung cancer	2/31	72/8223	0.029852167	0.074892278	0.042449065	STAT3/E2F1	2
hsa05133	Pertussis	2/31	76/8223	0.032978576	0.081309249	0.046086214	JUN/RELA	2
hsa05140	Leishmaniasis	2/31	77/8223	0.033779634	0.081872673	0.046405564	JUN/RELA	2
hsa04662	B cell receptor signaling pathway	2/31	84/8223	0.03959765	0.094374398	0.053491562	JUN/RELA	2
hsa04012	ErbB signaling pathway	2/31	85/8223	0.040458115	0.094844433	0.053757978	JUN/MYC	2
hsa05210	Colorectal cancer	2/31	86/8223	0.041325721	0.095315775	0.054025136	JUN/MYC	2
hsa04211	Longevity regulating pathway	2/31	89/8223	0.043970763	0.099806654	0.056570573	RELA/CREB1	2
hsa05417	Lipid and atherosclerosis	3/31	215/8223	0.046305232	0.10269128	0.058205584	JUN/STAT3/RELA	3
hsa04658	Th1 and Th2 cell differentiation	2/31	92/8223	0.046677855	0.10269128	0.058205584	JUN/RELA	2
hsa04912	GnRH signaling pathway	2/31	93/8223	0.047593724	0.103119735	0.058448433	JUN/EGR1	2
